# Inducing Efficient and Multiwavelength Circularly Polarized Emission From Perovskite Nanocrystals Using Chiral Metasurfaces

**DOI:** 10.1002/adma.202413967

**Published:** 2024-11-15

**Authors:** Nadesh Fiuza‐Maneiro, Jose Mendoza‐Carreño, Sergio Gómez‐Graña, Maria Isabel Alonso, Lakshminarayana Polavarapu, Agustín Mihi

**Affiliations:** ^1^ CINBIO Universidade de Vigo Department of Physical Chemistry Materials Chemistry and Physics Group Campus Universitario As Lagoas Vigo 36310 Spain; ^2^ Institute of Materials Science of Barcelona ICMAB‐CSIC Campus UAB Bellaterra 08193 Spain

**Keywords:** chirality, circularly polarized photoluminescence, field enhancement, nanoimprint, Perovskite nanocrystals

## Abstract

Chiral nano‐emitters have recently received great research attention due to their technological applications and the need for a fundamental scientific understanding of the structure‐property nexus of these nanoscale materials. Lead halide perovskite nanocrystals (LHP NCs) with many interesting optical properties have anticipated great promise for generating chiral emission. However, inducing high anisotropy chiral emission from achiral perovskite NCs remains challenging. Although chiral ligands have been used to induce chirality, their anisotropy factors (g_lum_) are low [10^−3^ to 10^−2^]. Herein, the generation of high anisotropy circularly polarized photoluminescence (CPL) from LHP NCs is demonstrated using chiral metasurfaces by depositing nanocrystals on top of prefabricated resonant photonic structures (2D gammadion arrays). This scalable approach results in CPL with g_lum_ to a record high of 0.56 for perovskite NCs. Furthermore, the differences between high‐index dielectric chiral metasurfaces and metallic ones are explored for inducing chiral emission. More importantly, the generation of simultaneous multi‐wavelength circularly polarized light is demonstrated by combining dielectric and metallic chiral metasurfaces.

## Graphical abstract

Circularly polarized emission of high dissymmetry (g_lum_ ≈ 0.56) is achieved from perovskite halide nanocrystals by depositing them on arrays of prefabricated resonant 2D gammadions. By using different materials, a hybrid photonic structure is managed to design with TiO_2_ and Au materials, in order to obtain resonances in the whole visible spectrum demonstrating the generation of multi‐wavelength circularly polarised light.

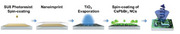

## 1 Introduction

Metal halide perovskite nanocrystals (NCs) are an appealing new type of semiconductors with remarkable optoelectronic properties such as high photoluminescence quantum yield, tunable emission across the visible spectrum, long charge‐carrier diffusion, and easy synthetic procedures. These exciting materials have potential applications in light‐emitting diodes (LEDs), single‐photon emission, photodetectors, lasers, and photovoltaics.^[^ ^1^, ^2^, ^3^, ^4^, ^5^ ^]^ In recent years, numerous studies have emerged attempting to induce new optical properties in metal halide perovskite NCs, being chirality highlighted as one of the most ambitious and interesting.^[^ ^6^, ^7^, ^8^, ^9^, ^10^, ^11^, ^12^, ^13^, ^14^, ^15^, ^16^ ^]^ The induction of chirality into LHPs can provide relevant properties such as circular dichroism (CD), spin polarization, and the emission of circularly polarized light (CPL).

Chiral NCs might have many applications with special mention for the areas of bio‐sensing,^[^ ^17^, ^18^, ^19^, ^20^ ^]^ bioimaging,^[^ ^21^, ^22^ ^]^ combating bacteria,^[^ ^23^, ^24^ ^]^ circularly polarized photocatalysis,^[^ ^25^, ^26^ ^]^ spintronics,^[^ ^27^, ^28^ ^]^ and phototherapy.^[^ ^29^, ^30^, ^31^ ^]^ In particular, chiral nano‐emitters are extremely interesting for energy‐efficient high‐definition displays and optical data communication. Some of the most frequently employed strategies to induce CPL from perovskite NCs involve the use of chiral capping ligands,^[^ ^12^, ^27^, ^32^, ^33^, ^34^, ^35^, ^36^, ^37^ ^]^ templated assembly of perovskite NCs into helical nanofibers^[^ ^38^, ^39^ ^]^ or chiral photonic architectures.^[^ ^40^ ^]^ The efficiency of CPL is typically quantified by the luminescence dissymmetry factor, g_lum_, a figure of merit obtained from the difference between the intensities of the two polarizations $\left(g_{\mathrm{lum}}=2\frac{I_{\mathrm{LCP}}-I_{\mathrm{RCP}}}{I_{\mathrm{LCP}}+I_{\mathrm{RCP}}}\right)$, where I_LCP_ and I_RCP_ are the intensities of the left‐ (LCP) and right‐ (RCP) circularly polarized components of the photoluminescence (PL), respectively. Despite great progress in the field of chiral perovskites, the luminescence dissymmetry factors (g_lum_) achieved so far are still relatively low to be implemented in actual devices. Chiral ligand‐capped NCs generally exhibit g_lum_ values in the range of 10^−3^–10^−2^.^[^ ^1^, ^9^, ^14^, ^41^, ^42^, ^43^ ^]^ Recently, nanophotonic structures sustaining chiral resonances showed great potential to endow circular dichroism to bulk perovskite films exhibiting an anisotropy factor of 0.49.^[^ ^44^ ^]^ Later on, similar 2D chiral architectures were used to impart chiral photoluminescence from perovskite NCs reaching g_lum_ values up to 0.15, which were further boosted to 0.3 with a high‐dielectric index coating (TiO_2_).^[^ ^40^ ^]^ In both cases, the perovskites were shaped into the chiral photonic architecture, requiring an additional fabrication step. In this work, we demonstrate that high dissymmetry chiral emission from achiral CsPbBr_3_ perovskites can be achieved by casting the nanocrystals directly on top of a prefabricated chiral metasurface. The chiral metasurfaces consist of square arrays of 2D gammadions fabricated on a resist by nanoimprinting lithography and coated with TiO_2_. The thickness of the titania layer was optimized to produce resonances matching the PL of the CsPbBr_3_ nanocrystals, thus reaching values of g_lum_ as high as 0.56 (see Figure S1, Supporting Information for experimental setup for CPL).

Furthermore, we explore the optical properties of metal‐coated chiral metasurfaces by evaporating Au instead of TiO_2_. The Au‐coated chiral structure exhibits a red‐shifted resonance compared with the TiO_2_‐coated gammadions, which can be used to achieve high emission dissymmetry factors of 0.4 in red‐emitting CsPbBr_1_I_2_ perovskite NCs. Finally, we demonstrate the generation of multicolor (green and red) chiral emission by combining the dielectric and metallic chiral metasurfaces into a single hybrid structure operating for both types of perovskite nanocrystals.

## 2 Results and Discussion

**Figure 1:**
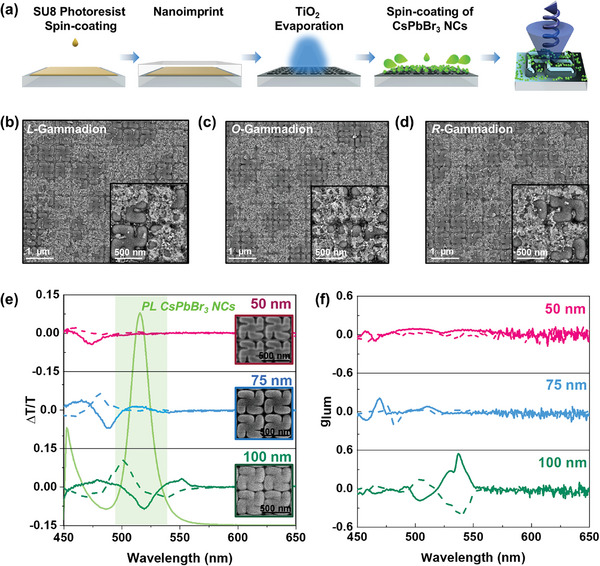
a) Scheme of the fabrication process of 2D‐chiral metasurfaces by soft nanoimprinting lithography of a photoresist followed by TiO_2_ thermal evaporation. The CsPbBr_3_ perovskite NCs are drop‐casted as a last step. b–d) SEM images of *L‐* (b), *O‐* racemic (c), and *R‐* Gammadions (d). e) PL spectra of CsPbBr_3_ perovskite NCs (green) and CD Transmission spectra of *L* (dotted) and *R* (solid) gammadions with different thicknesses of TiO2 (50, 75 and 100 nm) covered with CsPbBr_3_ perovskite NCs, whose PL overlaps with the resonance of 100 nm TiO_2_ (green shaded area). f) g_lum_ dissymmetry factors achieved for the TiO_2_ gammadions (50, 75, and 100 nm) with CsPbBr_3_ perovskite NCs.

The chiral nanostructures were fabricated by a nanoimprint lithography technique, as illustrated in **Figure** **1**a.^[^ ^45^, ^46^, ^47^ ^]^ First, a 100 nm layer of photoresist (Microchem, SU8 2000.5) was patterned via a hot‐embossing process on a previously oxygen plasma‐treated glass, using poly(dimethylsiloxane) (PDMS) molds pre‐patterned with a square array (period of 600 nm) of gammadions (500 nm in lateral size) covering a 9 mm^2^ patterned area. Metasurfaces with left (*L*), right (*R*), and racemic (*O*) orientations were fabricated. After printing, the samples were UV‐cured and annealed at 150 °C for 15 min to fix the structure. As previously reported, photonic structures of high‐index dielectric materials show improved interaction between light and matter, registering intense electromagnetic resonances.^[^ ^40^, ^48^, ^49^ ^]^ Here, we chose titania (TiO_2_) as the high refractive index material (n = 2.4) since it has no absorption in the visible range. We deposited different TiO_2_ coatings (50, 75, and 100 nm) onto the patterned resist films by e‐beam evaporation and compared their optical response in each case (Figure 1e,f). The transmission spectra for the chiral metasurfaces coated with different TiO_2_ thicknesses are shown in Figure S2 (Supporting Information), where a red‐shift is observed by increasing the thickness of the titania coating. Finally, the perovskite NCs were deposited on the surface by spin‐coating. The spin coating of the NCs onto the photonic surfaces is a fast process that did not allow for the self‐assembly of the nanocrystals as confirmed by SEM inspection.

The CsPbBr_3_ perovskite NCs used in this work were synthesized according to the typical ligand‐assisted ultrasonication method,^[^ ^50^ ^]^ obtaining a monodisperse colloidal solution with an average size of 15.08 nm and a characteristic photoluminescence emission peak centered at 519 nm. The colloidal dispersion was purified through centrifugation and redispersed in hexane (see Figure S3 (Supporting Information) for TEM images of the NCs), obtaining a solution that was subsequently spin‐coated over the TiO_2_ gammadions of various thicknesses. We need to consider that, to maintain the chiral optical features, we are required to optimize the thickness of the emitter layer. Besides, thick coatings could decrease the refractive index contrast and limit the extraction of polarized light by the chiral metasurface. The high quality of the perovskite‐coated 2D‐chiral metasurfaces with *R* (clockwise orientation), *L* (counterclockwise orientation), and *O* (racemic mixture of *R* and *L* gammadions) enantiomers was confirmed by scanning electron microscopy (SEM) imaging (Figure 1b–d; see additional images in Figure S4, Supporting Information).

**Figure 2:**
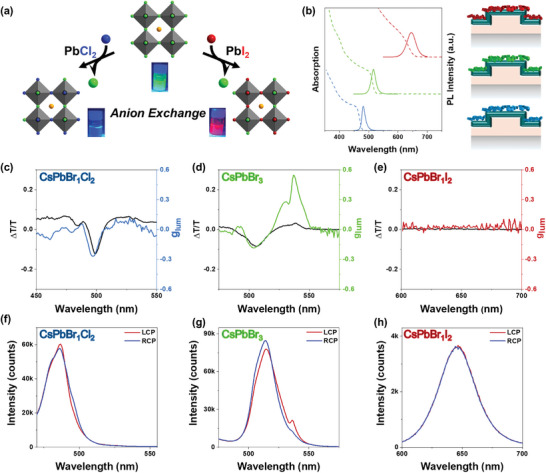
a) Anion exchange scheme of CsPbBr_3_ NCs with PbCl_2_ and PbI_2_. b) PL Intensity (solid line) and absorption spectra (dotted line) of CsPbBr_1_I_2_, CsPbBr_3_ and CsPbBr_1_Cl_2_ NCs. c–e) CD Transmission Spectra of 100 nm TiO_2_ coated Gammadions without perovskite NCs (black line) and g_lum_ dissymmetry factors of *L‐*TiO_2_ coated Gammadions with different perovskite NCs on top. (c) blue‐emissive CsPbBr_1_Cl_2_ NCs, (d) green CsPbBr_3_ and (e) red CsPbBr_1_I_2_ perovskite NCs. f–h) PL spectra of *L‐* TiO_2_ coated Gammadions with (f) blue‐emissive CsPbBr_1_Cl_2_ NCs, (g) green CsPbBr_3,_ and (h) red CsPbBr_1_I_2_ perovskite NCs.

The CsPbBr_3_ NCs addition onto the metasurfaces resulted in a red‐shift of the optical features with respect to the uncoated ones. Thus, the chiral resonance is shifted to lower energies after the perovskite deposition due to the change in the refractive index of the metasurface (Figure S2, Supporting Information). The figure of merit was calculated as $\Delta T/T=2\frac{T_{LCP}-T_{RCP}}{T_{LCP}+T_{RCP}}$. As shown in Figure 1f, a remarkable increase of the g_lum_ from 0.05 up to 0.56 is observed when increasing the TiO_2_ thickness for both *L‐* and *R‐* metasurfaces, due to a better overlapping of the emission band of the CsPbBr_3_ perovskite NCs (green shaded area Figure 1e) with the strong chiral resonance of the metasurface (see Figures S5, S6, Supporting Information for emission spectra from which Δ*T*/*T* were obtained). The uniformity of the sample is confirmed by obtaining equivalent results along various emission measurement spots (Figure S7, Supporting Information). Given that the chiral metasurface coated with 100 nm of TiO_2_ shows the strongest chiral resonances (Figure 1f; additional data can be found in Figure S7, Supporting Information), we explore its potential to polarize the emission of other perovskite compositions. The solutions of LHP NCs with different halide compositions were obtained through an anion exchange strategy by partially replacing the bromides with chloride or iodide anions.^[^ ^51^ ^]^ This allows us to effectively blue‐ or red‐shift the PL spectrum of the CsPbBr_3_, obtaining three different perovskite NCs covering the blue, green, and red parts of the electromagnetic spectrum (**Figure** **2**a,b). The synthesized mixed halide CsPbBr_1_Cl_2_ and CsPbBr_1_I_2_ perovskite NCs are nearly monodisperse with an average size of 12.18 and 11.57 nm, exhibiting PL peaks centered at 480 and 650 nm, respectively. All the perovskite NC solutions were purified through centrifugation and spin‐coated over the 100 nm‐thick TiO_2_ 2D‐chiral metasurface. The optical resonance present in the blue part of the spectrum leads to g_lum_ values of −0.2 (Figure 2c) for Cl‐Br mixed halide perovskites (*R‐*Gammadion) (*L‐*Gammadion characterization is shown in Figure S8, Supporting Information), which is not as high as the values observed for the CsPbBr_3_ NCs (Figure 2d) but it is still remarkably high, compared to values obtained with other strategies. This decrease of the g_lum_ signal for the mixed chloride/bromide perovskites can be explained by the fact that the blue resonance couples to a higher diffraction order, with less efficiency for the light extraction process. In the case of Br/I‐based red‐emitting perovskite NCs, the absence of chiral features in the transmittance spectra in the region correlates well with the absence of CPL signals. The results of these measurements effectively demonstrate that the presence of chiral resonances is required to effectively induce chiral emission from achiral LHP NCs by transferring the electromagnetic near‐field modes to the NCs placed near the photonic architecture (simulations of the mechanism of the chiral resonances can be found in Figures S9–S13, Supporting Information).^[^ ^52^ ^]^

**Figure 3:**
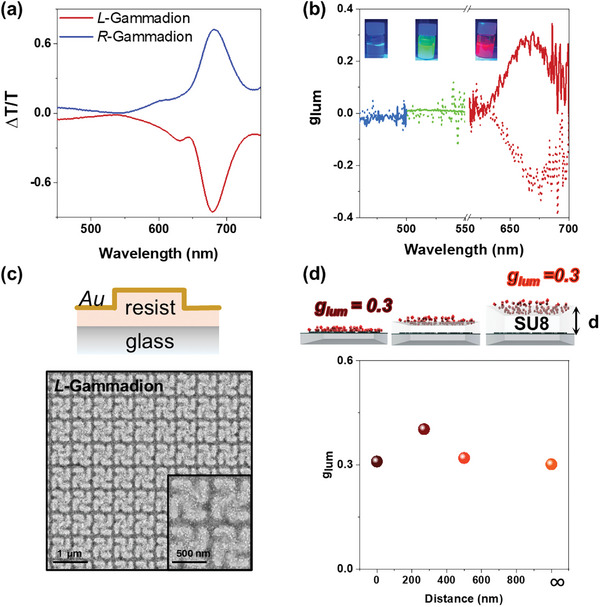
a) CD Transmission Spectra for *R‐* and *L‐* gammadions coated with 25 nm of Au. b) g_lum_ dissymmetry factors of 25 nm Au gammadions for blue CsPbBr_1_Cl_2_, green CsPbBr_3_, and red CsPbBr_1_I_2_ perovskite NC where the dotted line is *L‐* and the solid line is *R‐*gammadion, respectively. c) SEM high‐resolution images of Au‐coated *L‐*Gammadions with CsPbBr_1_I_2_ perovskite NCs. d) g_lum_ dissymmetry factors of *L‐* Au coated gammadions with different SU8 photoresist thicknesses increasing the distances of the chiral metasurfaces with respect to the red CsPbBr_1_I_2_ perovskite NC.

To extend the optical response of the metasurfaces to the red part of the spectrum without changing the geometry of the array, we considered other materials as possible coatings. In particular gold, due to its stability and inertness over time while presenting a plasmonic response. It should be noted that metallic metasurfaces can launch localized or surface plasmon polaritons at the emission wavelength, resulting in quenching or emission enhancement, depending on the arrangement of the emitters in the plasmonic cavity, as has been widely reported in the literature (For more extensive details, we recommend the reader to refer to Figures S14, S16, Supporting Information).^[^ ^53^, ^54^, ^55^ ^]^ A similar fabrication procedure to that previously employed was followed, evaporating 25 nm film of gold on the 2D gammadion instead of the dielectric material. As a result, the plasmonic chiral metasurface sustains a broad, red‐shifted resonance reaching ΔT/T up to 0.7 at 680 nm, suggesting a possibility of a better performance for generation of CPL from iodide‐based perovskite NCs (**Figure** **3**a). As predicted, the spectral matching of this resonance and the red‐emitting perovskite NCs emission band leads to high g_lum_ values up to 0.3 for CsPbBr_1_I_2_ NCs (Figure 3b; see Figure S15, Supporting Information for emission spectra). However, no remarkable g_lum_ values were observed for mixed chloride/bromide or bromide perovskites due to the absence of resonances in the region of their distinctive emission.

**Figure 4:**
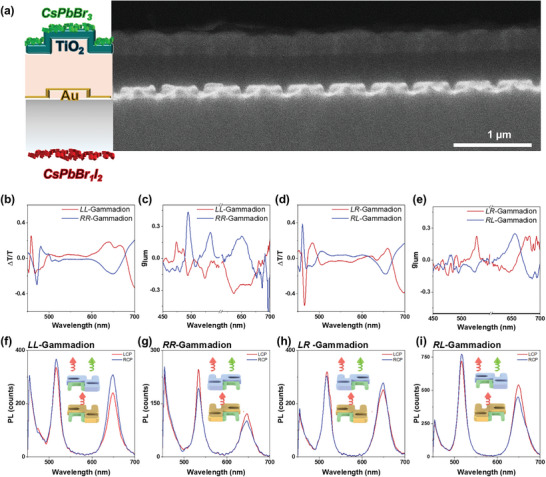
a) Scheme of Au 25 nm and TiO_2_ 100 nm hybrid chiral metasurfaces and SEM cross‐section. b) CD Transmission spectra for *LL*/*RR‐* hybrid gammadions. c) g_lum_ dissymmetry factors of *LL* and *RR‐* hybrid gammadions with CsPbBr_1_I_2_ and CsPbBr_3_ perovskite NCs. d) CD Transmission spectra for *RL*/*RL‐* hybrid gammadions. e) g_lum_ dissymmetry factors for hybrid structure with opposite handedness (*LR‐* and *RL*‐) with CsPbBr_1_I_2_ and CsPbBr_3_ perovskite NCs. f–i) PL characterization of (f) *LL‐* (g) *RR‐* (h) *LR‐* and (i) *RL*‐ gammadions.

To achieve chiral light emission covering the blue, green, and red parts of the spectrum using perovskite nanocrystals made of Cl, Br, and I, both titania and gold‐coated gammadion metasurfaces must be used in joint configuration. However, to avoid cross‐interference between the responses of the two chiral metasurfaces and to allow an easy integration, they must be spatially separated. Therefore, we explored the dissymmetric emission properties of the gold‐coated metasurface by varying its distance relative to the emitters by placing an intermediate dielectric spacer (SU8 resist) of different thicknesses between the chiral motifs and the emitting iodide‐based perovskite nanocrystals, as illustrated in Figure 3d (further information about the SU8 conditions to obtain different thickness is illustrated in Table S1, Supporting Information). High values of g_lum_ (≈0.3) were achieved for emitters placed even at hundreds of nanometers away from the metasurface (Figure 3d), leading to an almost distance‐independent g_lum_. These g_lum_ values were observed even if the red perovskites were placed on the backside of the glass substrate (1 mm thick), in an attempt to increase the distance from the emitters to the metasurface as far as possible. This result opens the possibility of exploring several emitters simultaneously by placing them spatially apart, overcoming the difficulties of mixing different perovskite NCs, which leads to anion exchange when placed in close proximity. Based on our findings, we designed a photonic architecture with the two chiral metasurfaces (TiO_2_ and Au) that could be used for the simultaneous generation of chiral light from green and red‐emitting perovskite NCs. It is worth noting that each architecture sustains chiral optical properties of different origins. Metallic chiral nanostructures couple light to plasmonic resonances showing extinction as nicely reviewed by Vogel et al.^[^ ^56^ ^]^ The fully dielectric chiral photonic structures do not show absorption and they exhibit dichroism by scattering off‐normal one of the two polarizations as described by Zhu et al.^[^ ^48^ ^]^ These results in the literature are in good agreement with our findings, briefly discussed in Figure S14 (Supporting Information). The first challenge was to obtain a photonic architecture sustaining chiral resonances both in the red and the green part of the spectrum. We designed a hybrid structure by stacking two gammadion metasurfaces (**Figure** **4**a): one coated with Au (25 nm) and the second covered with 100 nm TiO_2_ at ≈500 nm from the first metasurface, to avoid any coupling effect between them to obtain the resonances of both materials in their entirety^[^ ^57^ ^]^ (the resonances obtained for non‐separated hybrid structures are shown in Figure S17, Supporting Information). The second challenge was to employ perovskite nanocrystals with different compositions to enable green and red emissions while avoiding anionic exchange. Hence, we opted for placing the NCs at opposed sides of the substrate (Figure 4a): red perovskites were spin‐coated onto the back‐side of the glass substrate and green emissive ones are subsequently spin‐coated onto the TiO_2_ metasurface, separating the two types of perovskite NCs in space to avoid halide exchange between them. Upon excitation from the glass side with a 405 nm pulsed laser, the red unpolarized emission of CsPbBr_1_I_2_ is directed toward the plasmonic metasurface. This leads to the partial polarization of red emission due to a fraction of the LCP (for *L*‐handed) being absorbed by the structure. The transparency and lack of chiral resonances of TiO_2_ in the red emission band enables the partially polarized light to pass through the second metasurface without changing its polarization state. On the other hand, the green emissive CsPbBr_3_ NCs do not absorb the red‐emitted light due to their higher bandgap. Since both metasurfaces act independently, it is possible to select the preferential handedness that will be endowed to each color, *i.e*., green and red. To illustrate this, five different possible hybrid structures were fabricated by combining the gammadions of equal (Au/TiO_2_ *LL*/*RR*) or opposite (Au/TiO_2_ *LR*/*RL)* handedness (where the first letter corresponds to the first imprint coated with 25 nm Au and the next letter corresponds to the second one coated with 100 nm TiO_2_), as well as an achiral substrate by using racemic imprints (*OO*) (racemic imprint emission spectra are shown in Figure S18, Supporting Information). The created hybrid structures exhibit resonances at 480 and 650 nm, aligning spectrally with the isolated metasurfaces of gold and TiO_2_, as expected for the uncoupled system (see Figure 4f–i for the emission spectra). This is demonstrated in Figure 4b,d, depicting resonances for both matching (*LL*, *RR*) and opposite (*LR*, *RL*) handedness configurations. In the case of using the same handedness for both metasurfaces, the chiral resonances sustained present the same polarization sign of interaction, which is transferred to higher values of the same emitted polarizations of RCP (LCP) for the *L*‐Gammadion (*R*‐Gammadion) metasurfaces at 520 and 650 nm, as seen in Figure 4c (PL can be seen in Figure 4f,g). On the contrary, Figure 4e shows that if Au and TiO_2_ metasurfaces have opposed handedness, then the PL of green and red perovskite NCs shows an opposed sign (Figure 4h,i). The racemic mixtures did not show any CPL spectra as expected in the spectral range explored. In all the CPL spectra, a small contribution at 500 nm is noticed, coming from the TiO_2_ emission, which is further enhanced when calculating the g_lum_ values obtained at that wavelength.

## 3 Conclusion

In conclusion, we successfully achieved CPL with high g_lum_ values across the entire visible spectrum using the resonant interactions of green and red emissive perovskite NCs with chiral metasurfaces. We have gained insight into how the 2D‐chiral metasurface system transfers its chiral properties, being highly dependent on the material of which the chiral motif is composed (Au, TiO_2_). This enables the system to exhibit various resonances to induce chirality in a broad spectral range. The high g_lum_ values obtained using different coating materials showed a versatile and efficient approach to induce chirality in achiral perovskite NCs of mixed compositions at once in the entire visible range. Our findings shed light on the mechanism of chirality induction in perovskite CsPbBr_3_ NCs using TiO_2_‐coated chiral metasurfaces. The spectral matching of the emission wavelengths and the chiral resonances is fundamental to ensure the coupling of the emitters to the metasurface obtaining the highest g_lum_ values reported to date (up to 0.56). In addition, we fabricated gold‐coated chiral metasurfaces sustaining optical resonances at red wavelengths that can couple with the emission of iodide‐based perovskites. Finally, we combine both the dielectric and metallic metasurfaces to create a hybrid photonic architecture capable of polarizing the photoluminescence of perovskite nanocrystals with high dissymmetry luminescence factors throughout the entire visible range synchronously by placing the NC emitters of different colors at different spatial locations.

## Conflict of Interest

The authors declare no conflict of interest.

## Supporting information



Supporting Information

## Data Availability

The data that support the findings of this study are available from the corresponding author upon reasonable request.
