# Supplementary material for: Inducing Efficient and Multiwavelength Circularly Polarized Emission From Perovskite Nanocrystals Using Chiral Metasurfaces
Source: Adv Mater. 2024 Nov 15;36(52):2413967. doi: 10.1002/adma.202413967 (PMC11681301; doi:10.1002/adma.202413967)
Supplement: Supplementary file 1 — Supporting Information [file ADMA-36-2413967-s001.docx]

***Supporting Information***

**Inducing Efficient and Multiwavelength Circularly Polarized Emission from Perovskite Nanocrystals Using Chiral Metasurfaces**

Nadesh Fiuza-Maneiro, Jose Mendoza-Carreño, Sergio Gómez-Graña, Maria Isabel Alonso, Lakshminarayana Polavarapu,* and Agustín Mihi*

N. Fiuza-Maneiro, S. Gómez-Graña, L. Polavarapu

CINBIO, Universidade de Vigo, Department of Physical Chemistry Materials Chemistry and Physics Group, Campus Universitario As LagoasVigo 36310, Spain

E-mail: lakshmi@uvigo.es

J. Mendoza-Carreño, M. I. Alonso, A. Mihi

Institute of Materials Science of Barcelona ICMAB-CSICCampus UAB, Bellaterra 08193, Spain

E-mail: amihi@icmab.es

N. Fiuza-Maneiro and J. Mendoza-Carreño contributed equally to this work.

**Contents:**

**Supplementary Information 1. Relevant Magnitudes in Chirality Measurements**

**Supplementary Information 2. Experimental Section**

**Supplementary Information 3. CsPbX_3_ Nanocrystals Characterization**

**Supplementary Information 4.** **Transmission CD Characterization for TiO_2_ Gammadions**

**Supplementary Information 5. Low Magnification SEM Images of Gammadion Arrays**

**Supplementary Information 6. Experimental Transmission CPL Characterization of TiO_2_ Coated Gammadions**

**Supplementary Information 7. TiO_2_ 100 nm *L-*Gammadion Characterization**

**Supplementary Information 8. FDTD Simulations**

**Supplementary Information 9. Emission Spectra Obtained for 25 nm Coated Au Gammadions with Perovskite NCs of Different Compositions**

**Supplementary Information 10. Unraveling Possible PL Quenching Effects Depending on the Coating Material**

**Supplementary Information 11. Transmission CD of Hybrid Structures**

**Supplementary Information 12. PL Spectra Characterization of Racemic Hybrid Structure**

**Supplementary Information 1. Relevant Magnitudes in Chirality Measurements**

The fabricated coated chiral metasurfaces have been characterized using two spectroscopic techniques that aim to collect the photoluminescence in addition to the light transmitted through the sample, through an optical setup as illustrated in **Figure S1**.

**Figure S1a** schematically represents the experimental setup employed to measure the differential transmittance across the metasurface, therefore it renders a differential extinction spectrum (1-T) collected when illuminated with *L*- or *R*- circularly polarized incident light. This difference in transmitted light is due to the light that is absorbed or scattered by the chiral metasurface. In samples without scattering and considering negligible reflection, the difference in transmittance is due to the difference in molecular absorption of each polarization by the material, also known as circular dichroism (CD)^[1-3]^ as given in equation (1):

𝐶𝐷 = 𝑎_𝐿𝐶𝑃_ – 𝑎_𝑅𝐶𝑃_ (1)

Where the terms 𝑎_𝐿𝐶𝑃_ and 𝑎_𝑅𝐶_ are the L and *R*- absorbed circularly polarized light, respectively. Given the need to standardize this quantity, Tang and Cohen^[4]^ introduced the well-known dissymmetry factor or g-factor whose definition appears in equation (2):

$g=\frac{a_{LCP}-a_{RCP}}{\frac{1}{2}(a_{LCP}+a_{RCP})}$ (2)

In our case, this magnitude only takes absorption as a basis for differential transmittance.

For periodic arrays acting as scattering centers, these definitions underestimate the transmittance differences for L or RCP incident light. Circularly polarized light is obtained by a linear polarizer that is aligned at ±45° with the fast axis of a quarter-wave plate (**Figure S1a**). This light is incident on the chiral metasurface giving rise to differences in absorption, but also in scattering, which must be considered in the differential transmittance. Considering a metasurface that only scatters a circular polarization out of the propagation axis without absorbing its energy, we would still measure a transmittance difference on the normal axis that could be assumed as circular dichroism. Consequently, we have reconsidered the definitions of CD and dissymmetry factors to introduce not only absorption but also scattering processes, according to previous work in the literature on periodic arrays of chiral scatterers, through the normalization of the unpolarized transmittance:

CD =$T_{LCP}-T_{RCP}$ (3)

$g=\frac{{\Delta T}_{L-CP}}{\frac{1}{2}T_{unpolarized}}=\frac{T_{LCP}-T_{RCP}}{\frac{1}{2}T_{unpolarized}}$ (4)

The reported CD and dissymmetry factors have been calculated using the corresponding equations (3) and (4). Spectroscopic measurements of circularly polarized light emission have been generated directly from perovskite (PVK) nanocrystals deposited on chiral metasurfaces coated with various materials. The C4 symmetry characteristic of the gammadion structure results in negligible contribution of linear dichroism, so that only circularly polarized light results in a dissymmetry in the measured PL.

To perform this measurement, as shown in **Figure S1b**, the emitted circularly polarized light is passed through a superachromatic quarter-wave plate that will transform each handedness polarization into orthogonal linear polarization states. Then, both polarizations are sent to a Glan-Thompson linear polarizer that allows only the vertical polarization to pass so that the horizontal polarization is reflected at 90°, hence, a handedness polarization state is redirected away from the detection axis. Either polarization can be selected by positioning the quarter-wave plate at ±45° concerning the vertical axis of the linear polarizer.

In the same way as the circular dichroism dissymmetry factor, which denotes the dissymmetry in absorption or extinction processes for incident circularly polarized light, we can calculate the luminance dissymmetry factor, that quantifies the dissymmetry of the generated circularly polarized photoluminescence, which can be calculated according to equation (5) below^[5-7]^:

$g_{lum}=\frac{{\Delta I}_{L-R}}{\frac{1}{2}I}=2\frac{I_{LCP}-I_{RCP}}{(I_{LCP}+I_{RCP})}$ (5)

Where 𝐼_𝐿𝐶𝑃_ and 𝐼_𝑅𝐶𝑃_ are the emitted PL intensities for left and right circularly polarized luminescence and 𝐼 is the total PL intensity.


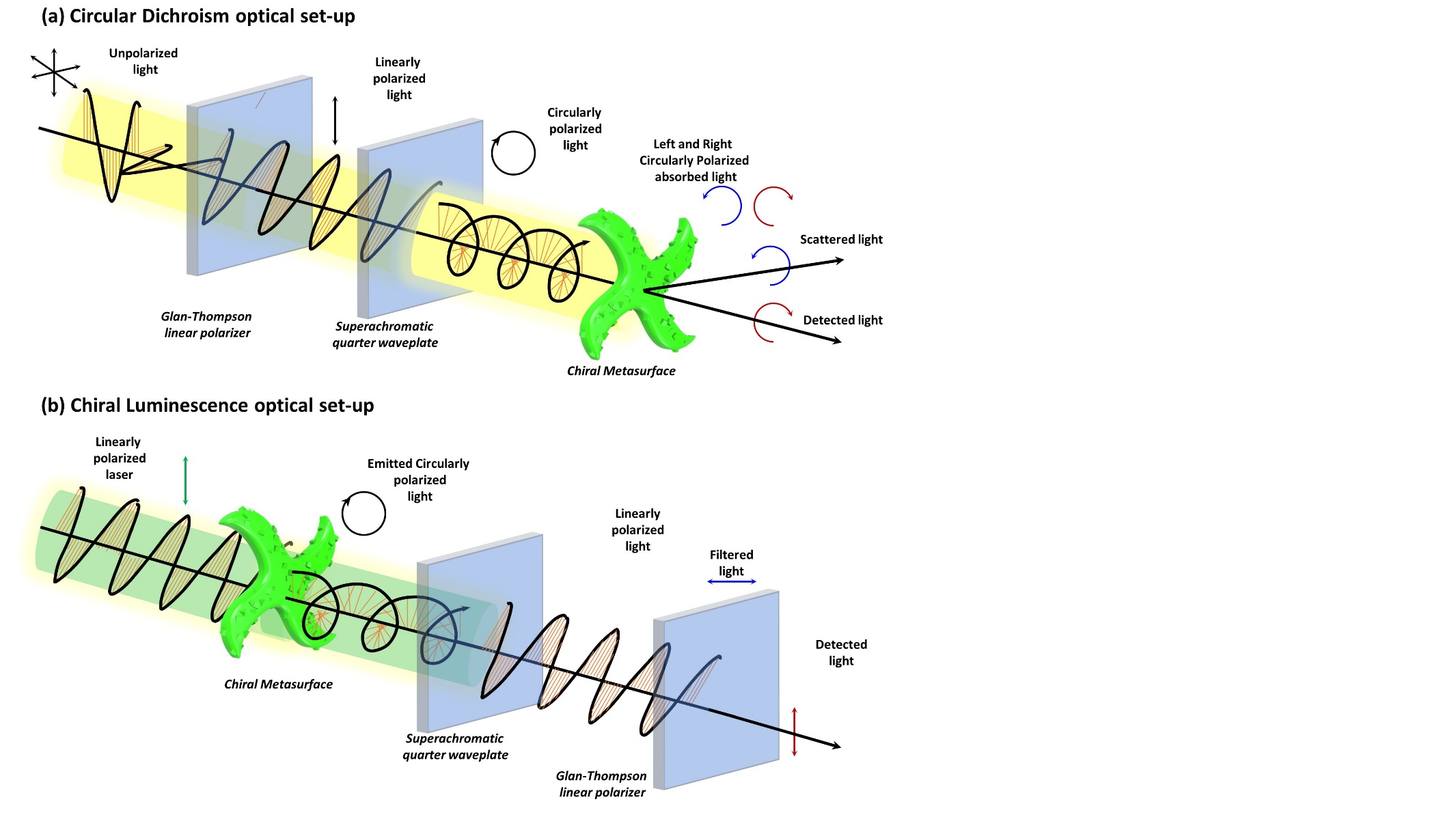


**Figure S1**. Experimental setup employed for (a) transmission CD measurements through the interaction between circularly polarized light with the chiral metasurface. (b) Circularly polarized emitted light measurements.

**Supplementary Information 2. Experimental Section**

*Materials:* Cesium carbonate (Cs_2_CO_3_, 99.9%), lead (II) bromide (PbBr_2_, >98%), lead (II) iodide (PbI_2_, 99%), 1-octadecene (C_18_H_36_, 90%), oleic acid (C_18_H_34_O_2_, 90%), and oleylamine (C_18_H_37_N, 70%) were purchased from Merck. SU8 Photoresin (14% in cyclohexanone) was purchased from Microchem. Hellmanex III solution and hexane solutions were purchased from Millipore-Sigma. Acetone, isopropanol, and NaOH were purchased from Labbox. PDMS (Sylgard 184) was purchased from Dow Corning (Michigan, USA). The hard PDMS (hPDMS) mixture kit was purchased from Gelest (USA). All chemicals were used as received.

*Preparation of CsPbBr_3_, CsPbBr_1_I_2_ and CsPbBr_1_Cl_2_ Perovskite NCs:*

*Synthesis of CsPbBr_3_ Perovskite NCs*: In a typical synthesis, 15 mL of octadecene, 1.5 mL of oleic acid, and 1.5 mL of oleylamine and the precursor powders (1 mmol of Cs_2_CO_3_ and 3 mmol of PbBr_2_) were loaded in a 50 mL glass vial. Then, the reaction medium was subjected to tip-ultrasonication (SONOPULS HD 3100, BANDELIN) at a power of 30 W for 30 min. Throughout the course of the reaction, a color change from colorless to yellow could be appreciated indicating the formation of perovskite NCs. The solution was purified by centrifugation (10,000 rpm, 10 min) and the pellet was redispersed in 6 mL of hexane. Then, a second centrifugation stage was carried out (5,000 rpm, 10 min) in order to remove the large particles. The supernatant was collected and used after another dilution for the obtention of mixed halide perovskite NCs.

*NCs by Halide Exchange Using PbI_2_ and PbCl_2_ Solution.*

*Preparation of the PbX_2_ Solution*: 1 mmol of PbI_2_ or PbCl_2_, 1.5 mL of oleylamine, 1.5 mL of oleic acid, and 100 mL of hexane were loaded in a 250 mL Pyrex bottle. The solution was heated-up until 60 °C under vigorous stirring until the salt was completely dissolved. The PbI_2_ or PbCl_2_ solution in hexane was employed as iodide and chloride source for the synthesis of mixed halide perovskite NCs.

*Synthesis of CsPbBr_1_I_2_ and CsPbBr_1_Cl_2_ Perovskite NCs*: CsPbBr_1_I_2_ and CsPbBr_1_Cl_2_ NCs were synthesized by halide exchange reaction. In a typical synthesis, 500 µL of the CsPbBr_3_ NCs colloidal dispersion previously synthesized were loaded in a 25 mL glass vial, adding 1.5 mL of hexane. Then, the PbI_2_ or PbCl_2_ solution was consecutively added under vigorous stirring until the PL tuned to the desired wavelengths (650 and 480 nm). The reaction was monitored by PL, observing a shift in the PL emission that indicated the halide exchange.

*Preparation of the Patterned Stamps*: *Preparation of the Original Master Structures*: The original silicon masters (purchased from CONSCIENCE, Sweden) were constituted of arrays of gammadion shapes with 500 nm width and 120 nm depth, disposed in a squared arrangement forming a lattice of 600 nm pitch in 3 × 3 mm^2^ areas. The masters were silanized with an anti-sticking layer of perfluorooctyl–trichlorosilane to prevent the adhesion of the silicones and resistance during replication. The silanization took place through chemical vapor deposition, leaving the masters for 30 min in a desiccator under vacuum together with 4 μL of perfluorooctyl–trichlorosilane. The substrates were rinsed with acetone and heated to 150 °C for 30 min to remove unreacted silane.

*Preparation of the Working Masters*: Intermediate masters were negative replicas of the original masters, used to obtain a final replica in PDMS of the original hole arrays. These negative hard molds were prepared using UV-nanoimprinting. Specifically, a drop of Ormostamp, a photosensitive resist, was placed directly on top of the silanized silicon master. Then, a cleaned glass slide was gently pressed on top making sure no bubbles remained trapped between the slide and the Ormostamp. The photoresist was then cross-linked and hardened under UV lamp for 5 min. To demold the Ormostamp working master, the substrates were placed on a hot plate at 160 °C. The difference in thermal expansion between photoresist and silicon induced the detachment of the Ormostamp mold. Finally, the working masters were silanized using isopropanol as solvent instead of acetone, to avoid the detachment of the photoresist layer from the glass slide.

*Preparation of the PDMS Molds*: Hybrid hard/soft PDMS molds were needed to ensure the correct replica of the working masters avoiding structural collapsing during demolding. In particular, they were composite molds where the structured layer (with a thickness of few micrometers) was made of hPDMS, while the backbone (several millimeters) was made of standard soft PDMS. To prepare the hPDMs mixture, 0.85 g of 7–8% vinylmethylsiloxane was mixed with 25 μL of 1,3,5,7 tetracetylcyclosilane, 3 μL of Pt catalyst, 250 μL g of 25–35% hydroxyl siloxane, and 750 μL g of toluene in this order. All the additions needed to take place under vigorous stirring. After 5 min of stirring, the obtained mixture could be used for ≈20 min, before it solidified after toluene evaporation. While it was still liquid, the mixture was drop-cast onto the master and spread over the surface using an air gun. This step ensures that the mixture covers entirely all the structures and leaves no microbubbles trapped. After repeating this last step two or three times for each working master, the substrates were left 1 h at room temperature to ensure complete evaporation of the toluene, and 1 more hour at 60 °C to cure the hPDMS. Next, the backbone was prepared by standard PDMS protocols. A 10:1 mixture of the monomer and curing agent were mixed vigorously and left degassing for approximately an hour. Then, the mixture was gently poured onto the samples with the cured hPDMS. Finally, the polymer was cured at 150 °C for another hour. Once the PDMS was cured, it was manually demolded from the master.

*Preparation of the Substrates for the Assembly*: Labbox neutral borosilicate microscope glass slides with high chemical resistance were used as substrates (standard line (26x76 mm)). The substrate preparation protocol comprised cleaning with acid piranha H_2_SO_4_: H_2_O_2_ 3:1 for 30 min. Then, they were rinsed with abundant distilled water and hydrophilized 5-10 min with O_2_ plasma cleaner.

*Nanoimprint Lithography*: Gammadion chiral metasurfaces were prepared by spin- coating 50 µL of the SU8 photoresin 5%w in cyclohexanone on top of a hydrophilized glass at (ω= 5000 rpm, α= 5000 rpm, 30s). After that, it was then covered with a prepatterned PDMS mold with the corresponding array and the pattern was imprinted through hot embossing at 100 ºC. Then, the mold was removed, and the structure was fixed under UV-light for 10 min. The structure was then finished by leaving it for 30 minutes at 150 ºC on a hotplate.

*High-Refractive-Index Coating*: 75 and 100 nm of TiO_2_ were deposited on top of the chiral metasurfaces by electron beam deposition (ATC-8E Orion from AJA International Inc.). 25 nm of Au were deposited on top of the structured SU8 gammadions arrays by thermal evaporation at a rate of 0.6 A/s.

*Preparation of the Chiral Metasurfaces:* CsPbBr_3_ NCs (1.5-0.2 mg/mL) were diluted in hexane in a relation 1:5 for further spin-coating. The various colloidal solutions of mixed halide perovskite NCs were prepared for spin-coating by cleaning from the excess surfactant by centrifuging at 14 000 rpm and concentrated 7.5 times in hexane for CsPbBr_1_Cl_2_ and CsPbBr_1_I_2_, respectively. Then, they were spin-coated over the sample (ω= 2000 rpm, α= 1000 rpm, 30s). Spin-coating the NCs onto the photonic surfaces is a fast process that did not allow for the self-assembly of the nanocrystals as confirmed by SEM inspection.

*Preparation of the Chiral Hybrid Metasurfaces:* Hybrid structures were fabricated following the previous procedures by SU8 imprinting and Au thermal evaporation (25 nm). Subsequently, another layer of SU8 (14%, ω= 2000 rpm, α= 2000 rpm, 30s) was added. Then, 100 nm of TiO_2_ were thermally evaporated. Finally, CsPbBr_1_I_2_ perovskite NCs were spin-coated on the backside of the sample and pure CsPbBr_3_ NCs on the top (ω= 2000 rpm, α= 1000 rpm, 30s).

*Preparation of SU8 Spacer Layers of Different Thicknesses: Spin-coating Conditions*

The spin-coating conditions used in the experiments to obtain different photoresist thicknesses are detailed below. For conventional Au and TiO_2_ gammadions, conditions (1) were used. However, in the experiments to study the influence of the proximity of the emitter to the chiral metasurface carried out for gold, conditions 2 to 4 were used. Finally, the construction of the chiral hybrid structure was carried out using conditions 1, with subsequent deposition of 25 nm of gold, followed by spin-coating of the higher concentration of photoresist with lower spin-coating rates (conditions 4) to obtain a thicker SU8 layer on which to perform the TiO_2_ deposition.

**Table S1.** SU8 Spin-coating conditions to obtain different thicknesses of the photoresist.

|  | **SU8** | **ω** | **α** | **Thickness** |
| --- | --- | --- | --- | --- |
| **1** | 5% | 5k | 5k | 100 nm |
| **2** | 5% | 6.5k | 6.5k | 70 nm |
| **3** | 10% | 2k | 2k | 270 nm |
| **4** | 14% | 2k | 2k | 470 nm |

*Optical Characterization*: *UV–Vis and Photoluminescence Spectroscopy*:

UV–vis extinction spectra were obtained using a Cary-60 UV–vis spectrophotometer (Agilent). PL spectra were obtained with a Cary Eclipse Fluorescence Spectrophotometer (Agilent). Quartz cuvettes with an optical path length of 1 cm were used for both optical analyses.

*Transmittance Circular Dichroism*: The optical measurements were carried out in a custom-made optical setup. A white tungsten halogen lamp (Ocean Optics, HL-2000-HP, FL, USA) corrected with two filters in the UV and NIR (Edmund Optics, SCHOTT BG64, and Thorlabs, SRF11) was coupled to a protected silver reflective collimator (RC08SMA-P01, Thorlabs) as light source injection. The light collection consisted of another protected silver reflective collimator coupled to a spectrometer (Ocean Optics, QEPro-FL) by an optical fiber. The collimated white light beam was sent through a Glan-Thompson Calcite Polarizer (GTH10M, Thorlabs) mounted on a stepper motor rotation mount (K10CR1/M, Thorlabs). The linearly polarized light obtained was directed to a superachromatic quarter wave-plate (SAQWP05M-700, Thorlabs) mounted at *π*/4 compared to the polarization direction on a rotation mount (ELL14, Thorlabs) to obtain a circularly polarized light beam. All the optical elements were controlled automatically by custom software (LabView NXG) to ensure the reproducibility of the measurements. The illumination area was controlled by a pinhole (SM1D12). Two dual-position sliders (ELL6, Thorlabs) were used to place a shutter and the sample in the beam path. The first one was used to measure the dark current of the spectrometer, whereas the second one was used to place the sample in the light beam. The sample was placed between a pair of 4× objectives (NA = 0.1).

*Chiral Emission Characterization*: A laser source (NPL41B, Thorlabs with peak emission at 405 nm was used to excite the perovskite NCs. The PL obtained from the samples was collected through a 4× objective with 0.1 NA and collimated to a superachromatic quarter-wave plate and a Glan-Thompson polarizer. Using a trigger controlled externally by an Arduino board (Arduino Uno), a fixed number of laser pulses were sent onto the sample to ensure both polarizations were given the same amount of energy in the excitation process. The laser signals were optically filtered using a long pass band filter (FELH0450, Thorlabs for 405 nm laser).

**Supplementary Information 3. Transmission CD Characterization**

Transmission CD characterization for the samples has always been carried out before the addition of the perovskite NCs and afterwards. However, for ease of understanding, we only give an explanatory example of how it is modified after the addition of the NCs. As can be seen in the figure below, the transmission CD signals decrease considerably in intensity and there is also a redshift in their resonance wavelength.


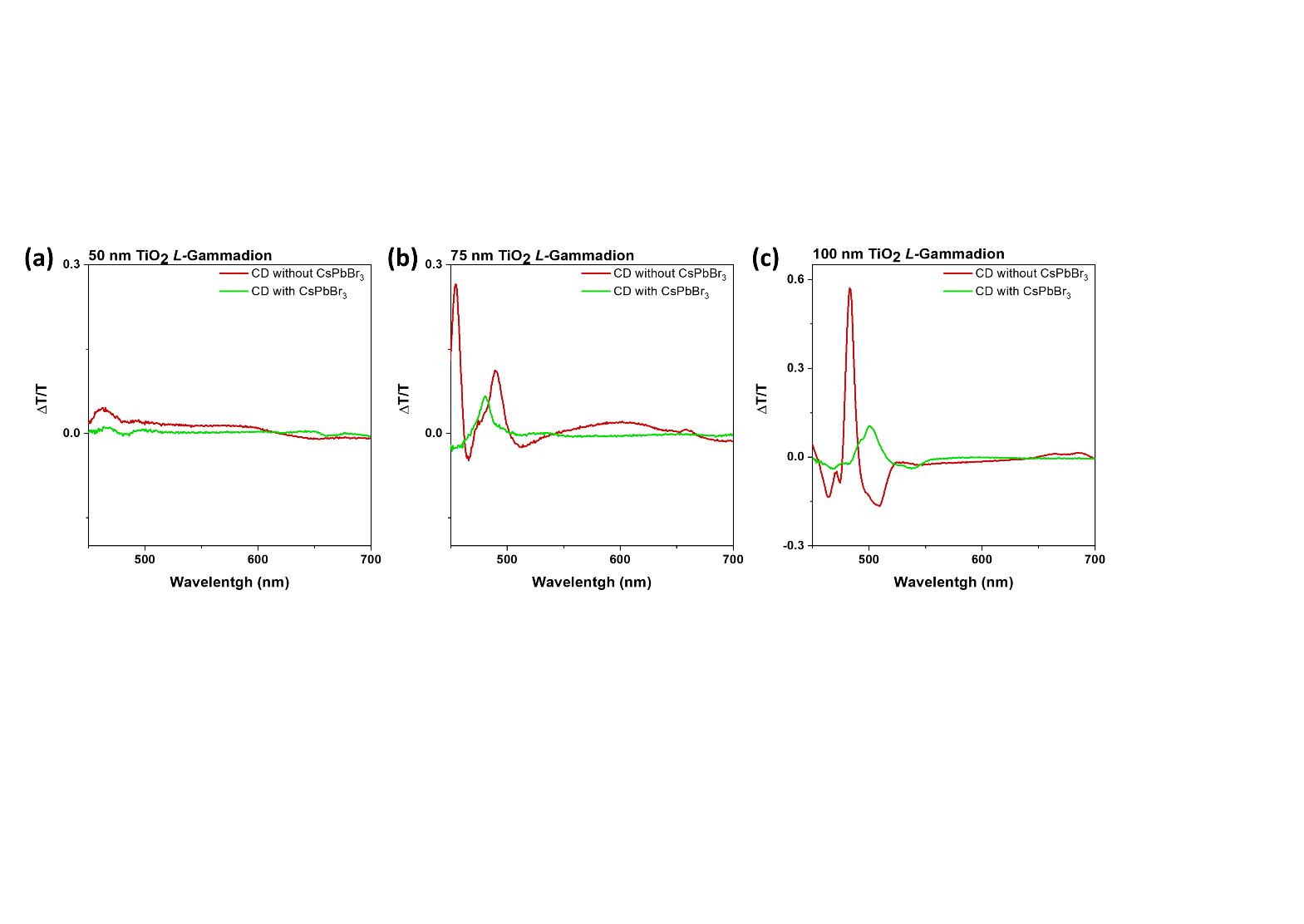


**Figure S2.** Differential transmittance factor spectra, ΔT/T , which consists of the difference of left and red handed transmittance as given in the formula $\Delta T/T=2\frac{T_{LCP}-T_{RCP}}{T_{LCP}+T_{RCP}}$ , before (red line) and after adding CsPbBr_3_ NCs (green line) on top of (a) 50 nm TiO_2_ coated gammadions, (b) 75 nm TiO_2_ coated gammadions, (c) 100 nm TiO_2_ coated gammadions. Increasing the TiO_2_ coating results in a redshift of the optical resonances and the Perovskite coating reduces the refractive index contrast of the system thus decreasing the magnitude of the resonances.

**Supplementary Information 4.** **CsPbX_3_ Nanocrystals Characterization**

The CsPbBr_3_ NCs obtained by ligand-assisted tip-ultrasonication are highly monodisperse. The colloidal dispersion was purified by two centrifugation steps. The green CsPbBr_3_ perovskite NCs exhibited photoluminescence (PL) with a maximum at 519 nm with a Gaussian FWHM of 18 nm. Red CsPbBr_1_I_2_ perovskite NCs exhibited PL with a maximum at 645 nm and the blue CsPbBr_1_Cl_2_ NCs exhibited a PL centered around 480 nm. The optical density of the NC dispersion reveals a small enough exciton binding energy so that exciton states and continuum onset overlay.


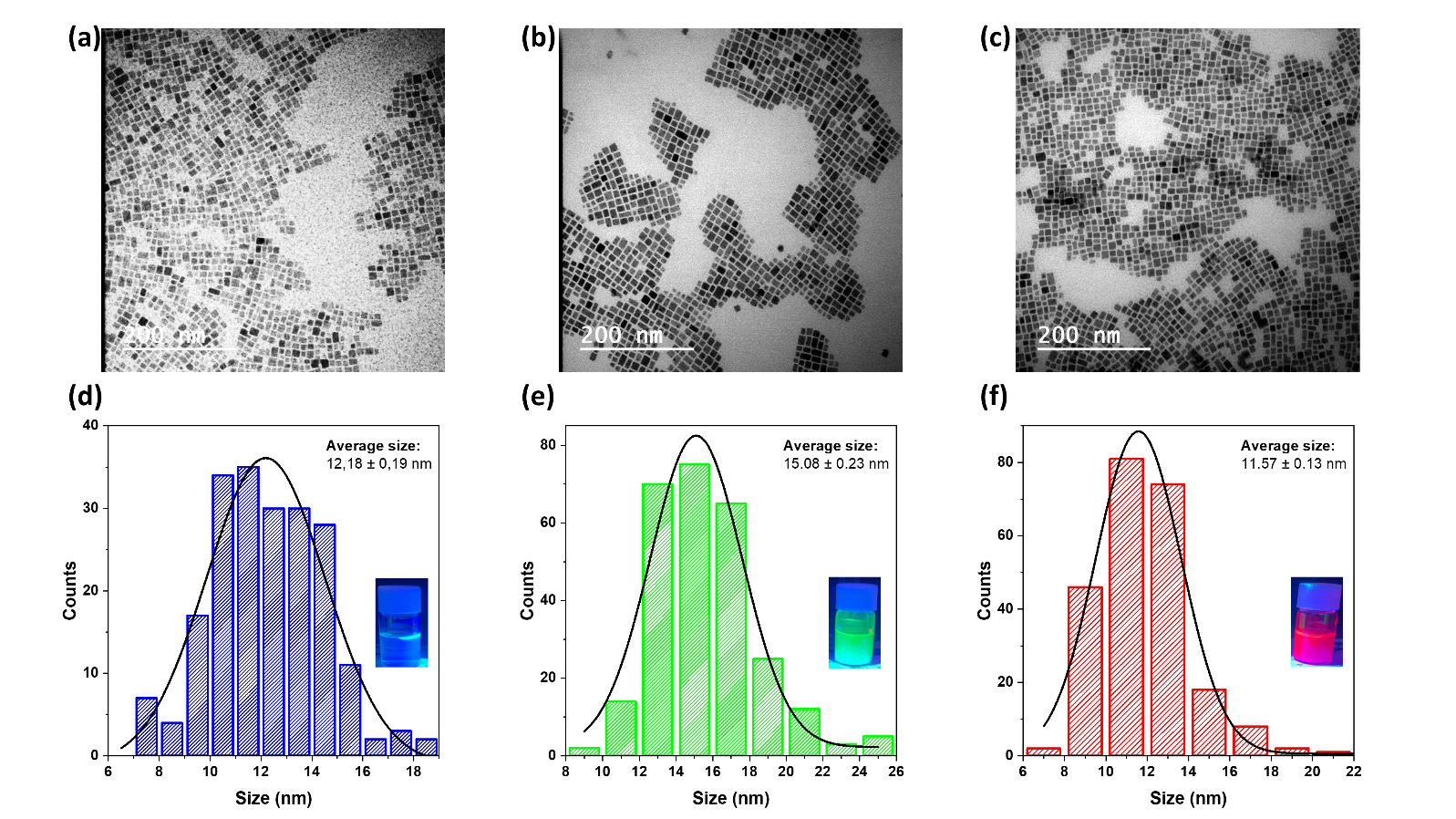


**Figure S3** Low magnification TEM images of the colloidal dispersions in hexane of (a) blue emissive CsPbBr_1_Cl_2_ NCs (b) green emissive CsPbBr_3_ NCs and (c) red emissive CsPbBr_1_I_2_ NCs. Size distribution histogram of (d) CsPbBr_1_Cl_2_ NCs (e) CsPbBr_3_ and (f) CsPbBr_1_I_2_ NCs.

**Supplementary Information 5. Low Magnification of Gammadion Arrays with Different Thickness**

The chiral metasurfaces obtained by nanoimprinting and coated with different thicknesses of TiO_2_ or Au through electron beam deposition or thermal evaporation respectively, show high homogeneity at low magnification SEM.


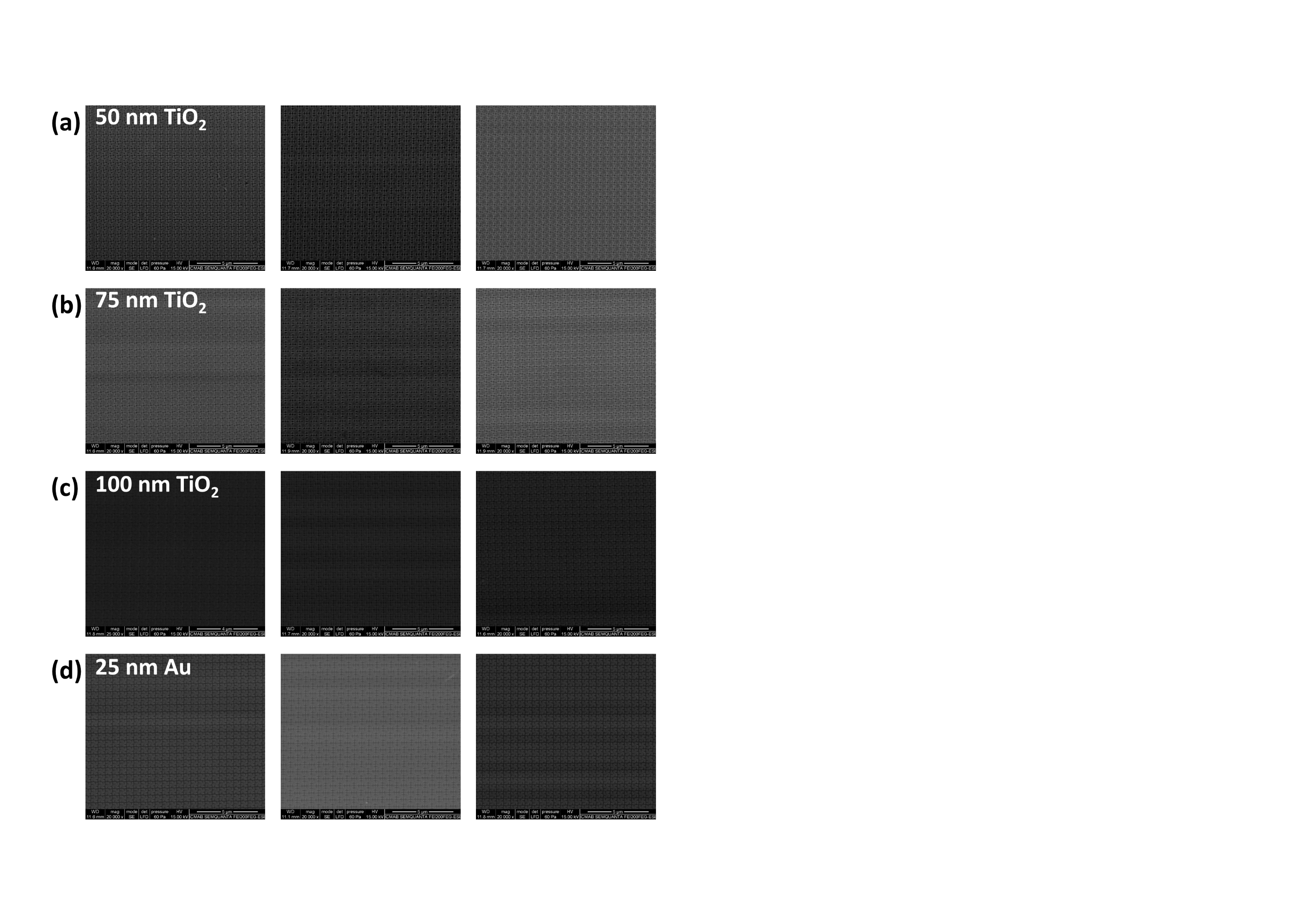


**Figure S4.** Low magnification images of gammadion arrays with different overlayer coatings in SEM. (a) 50 nm of TiO_2_ coated *L*-/*O*-/*R*- Gammadions. (b) 75 nm of TiO_2_ coated *L-*/*O-*/*R*- Gammadions. (c) 100 nm of TiO_2_ coated *L*-/*O*-/*R*- Gammadions. (d) 25 nm of Au-coated *L*-/*O*-/*R*- Gammadions.

**Supplementary Information 6. Experimental Transmission CPL Characterization of TiO_2_ Coated Gammadions**

*CPL of 50, 75, and 100 nm TiO_2_ Gammadions with CsPbBr_3_ PVK NCs*

First, we used different thicknesses of titania coating with perovskites of CsPbBr_3_ composition, and we could see how there is a greater difference between both polarizations for 100 nm of titania due to a better coupling between the resonance and the emission wavelength of the perovskites of this composition.


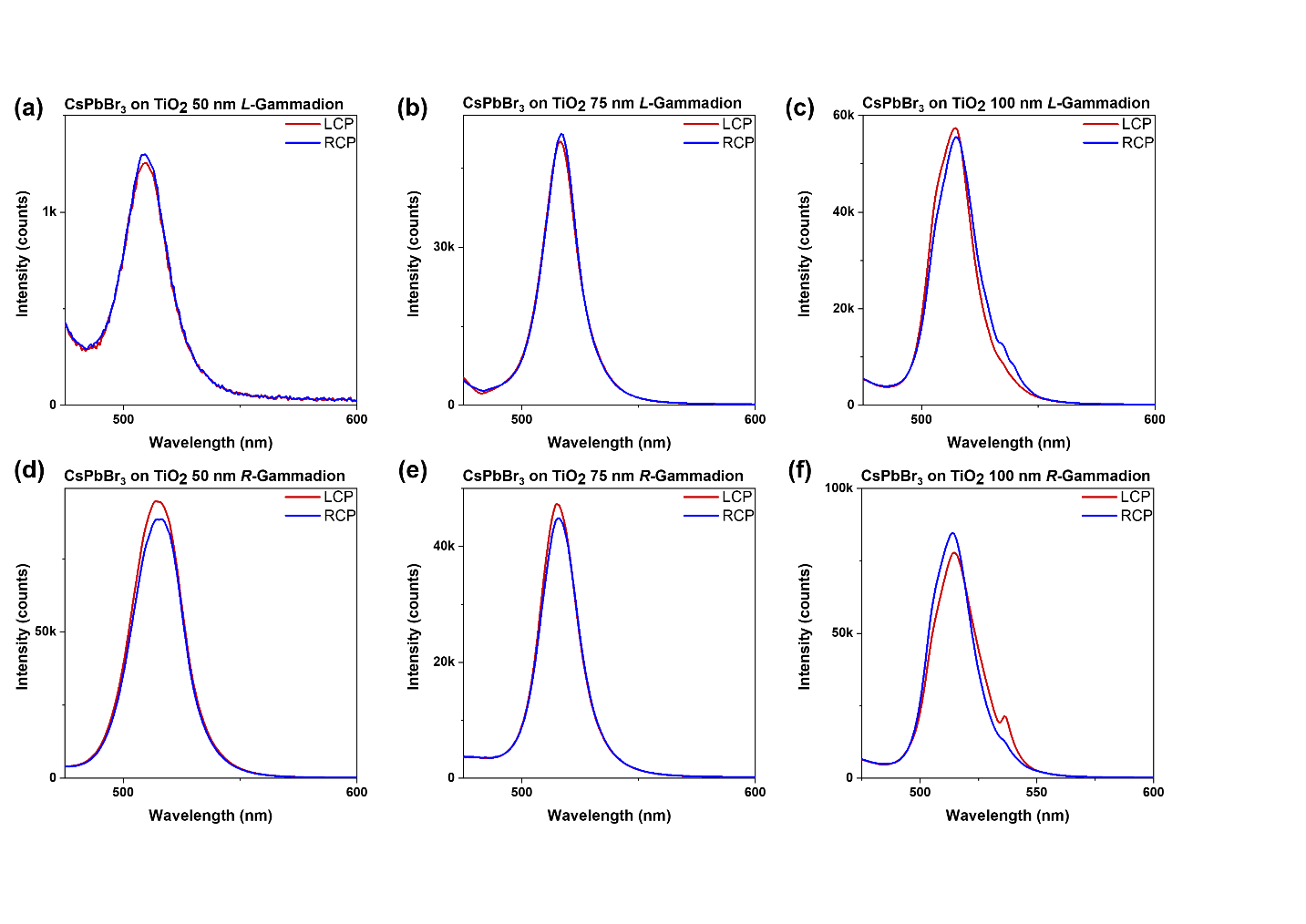


**Figure S5.** CPL spectra for *L-/R-*Gammadions with CsPbBr_3_ NCs on 50 nm (a,d), 75 nm (b,e) and 100 nm TiO_2_ coated gammadions (c,f).

Below we show the PL spectra of the different experiments carried out, in which a difference between the left and right circularly polarized light can be noticed in the cases where the emission wavelength matches with the resonance of the chiral metasurface.

As a control, off-pattern sample measurements were carried out along with racemic pattern samples for each of the cases. For the sake of simplicity and clarity, we do not include all performed measurements in the text and show a specific example below in which it is visualized how both polarizations show the same intensity.


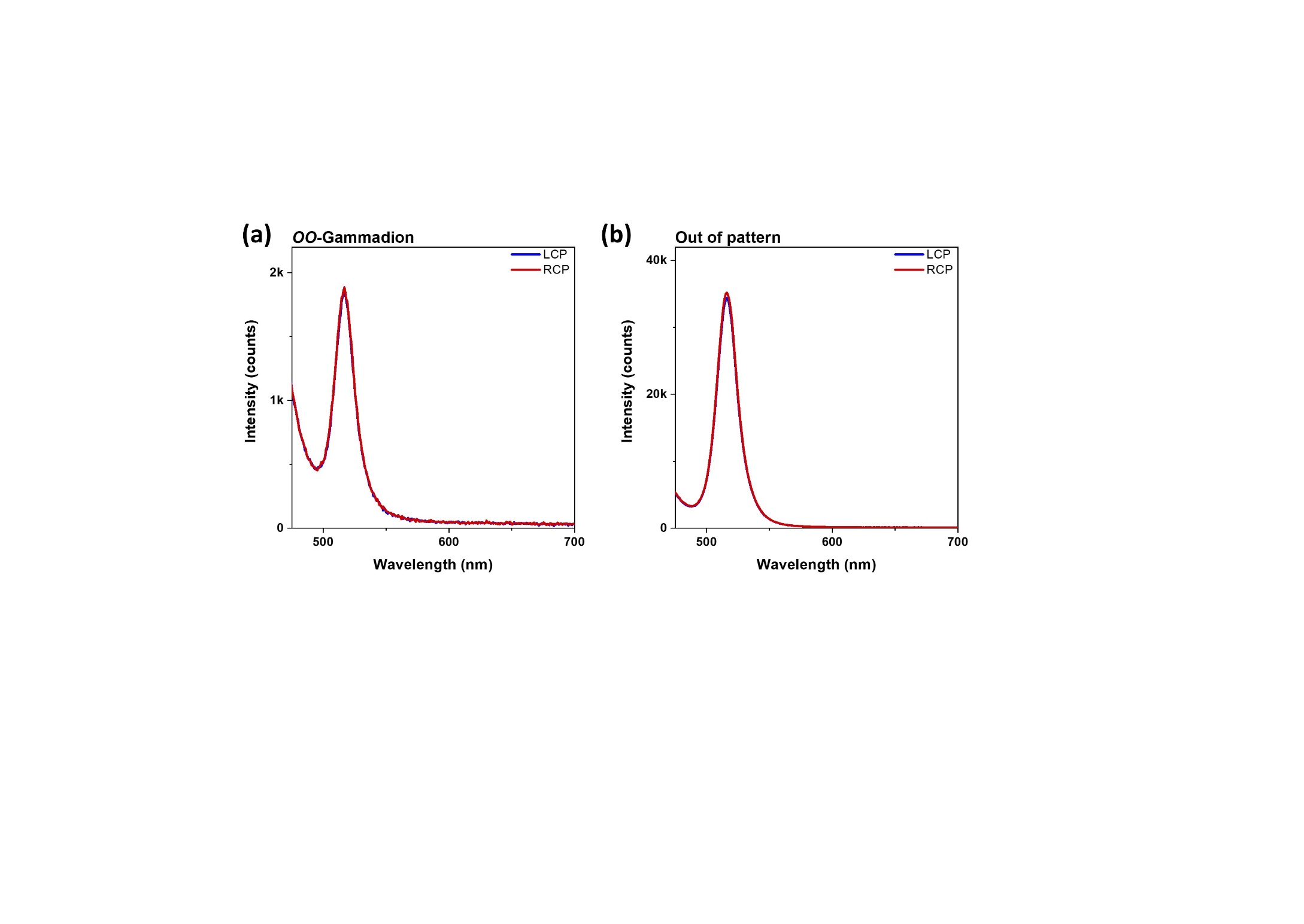
**Figure S6.** CPL for CsPbBr_3_ perovskites on 100 nm TiO_2_ chiral metasurfaces for racemic sample (a) and measuring out of pattern (b).

Since the perovskite NCs are not completely homogeneously distributed on the chiral metasurfaces, for each of the samples numerous measurements have been carried out and consistency in the data can be seen. However, small differences can arise as can be seen in the graphs below for three measurements taken for the 100 nm TiO_2_ coated gammadions drop-casted with CsPbBr_3_ NCs.


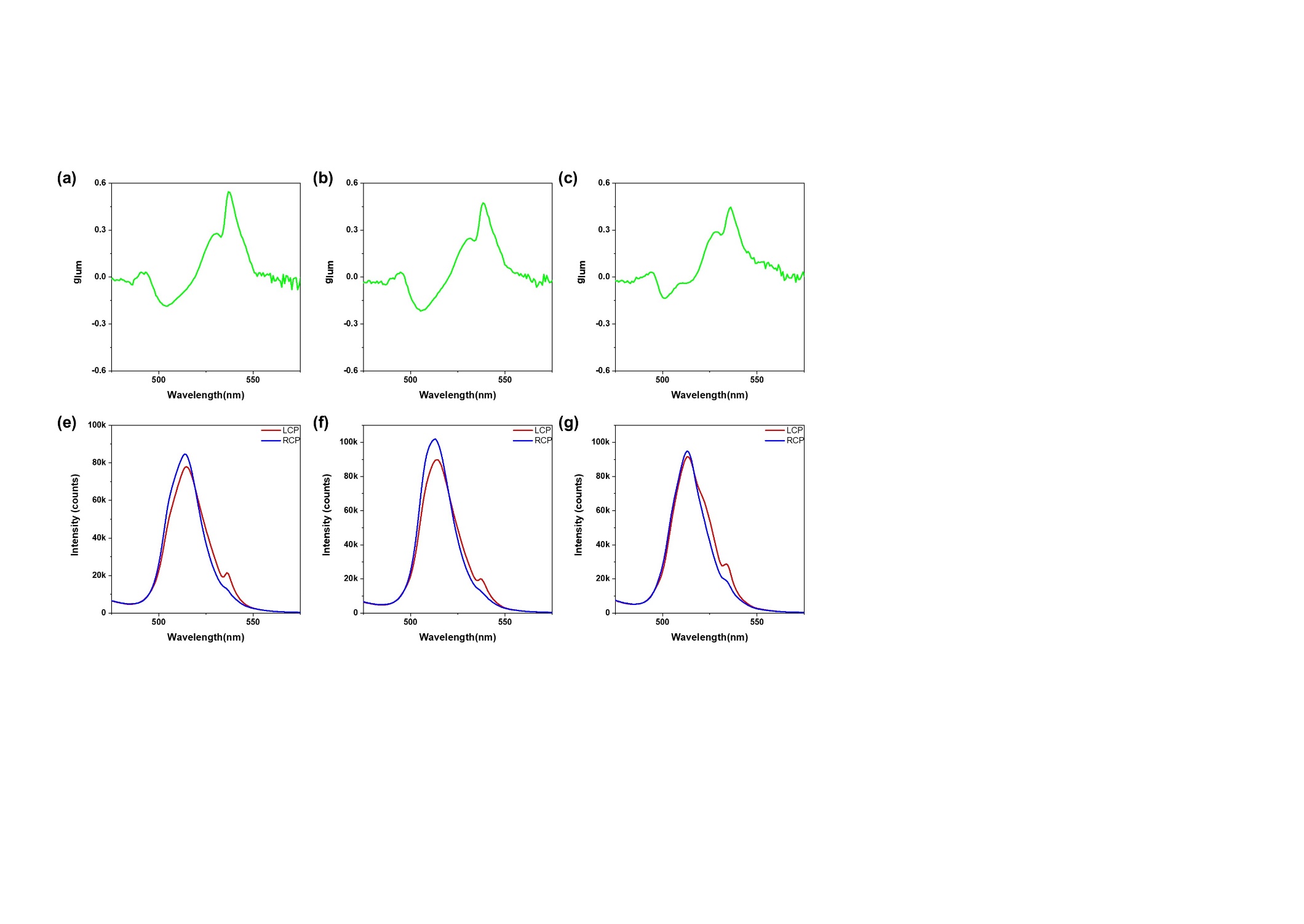


**Figure S7.** a-c) g_lum_ of 100 nm TiO_2_ coated *R-*Gammadions with CsPbBr_3_ NCs on top measured in different spots of the same sample. e-g) CPL of 100 nm TiO_2_ coated *R-*Gammadions with CsPbBr_3_ NCs on top measured in different spots of the 3x3 mm^2^ sample, where we measured in the center and at both sides.

**Supplementary Information 7. TiO_2_ 100 nm *L-*Gammadion Characterization**

Below, we can see the characterization carried out for the 100 nm TiO_2_-coated *L-*Gammadion with the perovskites of different compositions. We can appreciate in agreement, a polarization inversion concerning the enantiomer, obtaining again according to previous data, a higher g_lum_ value for the CsPbBr_3_ NCs as we expected. Lower values were obtained for the mixed Cl/Br perovskites as the emission does not coincide with the resonance, and no signal for the iodine perovskites as they do not have resonances in this region.


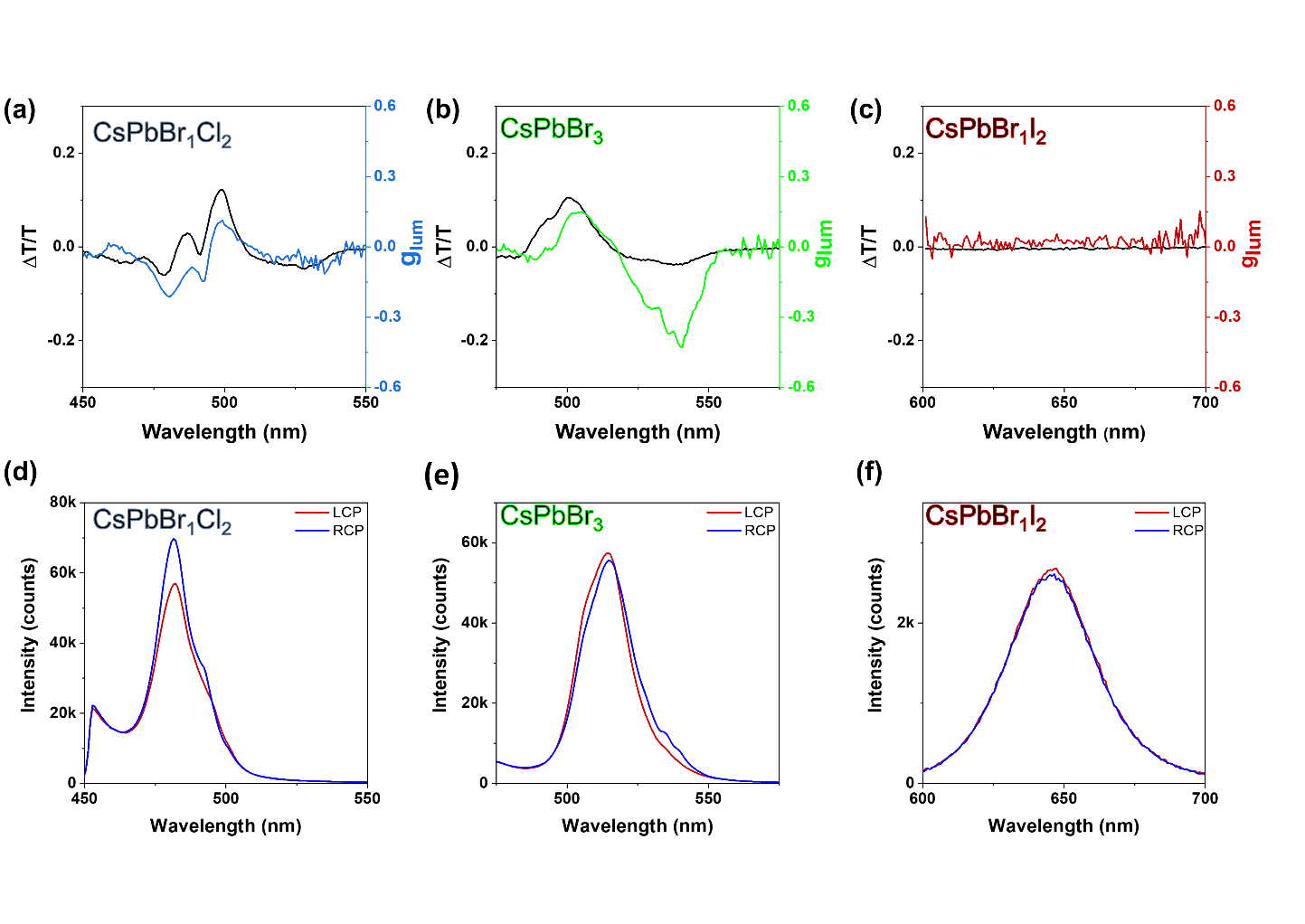


**Figure S8.** TiO_2_ 100 nm coated *L-*Gammadion transmission CD and g_lum_ characterization. CD transmission spectra of (a) CsPbBr_1_Cl_2_ (b) CsPbBr_3_ and (c) CsPbBr_1_I_2_ perovskite NCs. CPL characterization for (d) CsPbBr_1_Cl_2_ (e) CsPbBr_3_ and (f) CsPbBr_1_I_2_ perovskite NCs.

**Supplementary Information 8. FDTD simulations**

*FDTD Simulations: General Considerations:* FDTD simulations were performed using a commercial software (Lumerical Inc. by Ansys).


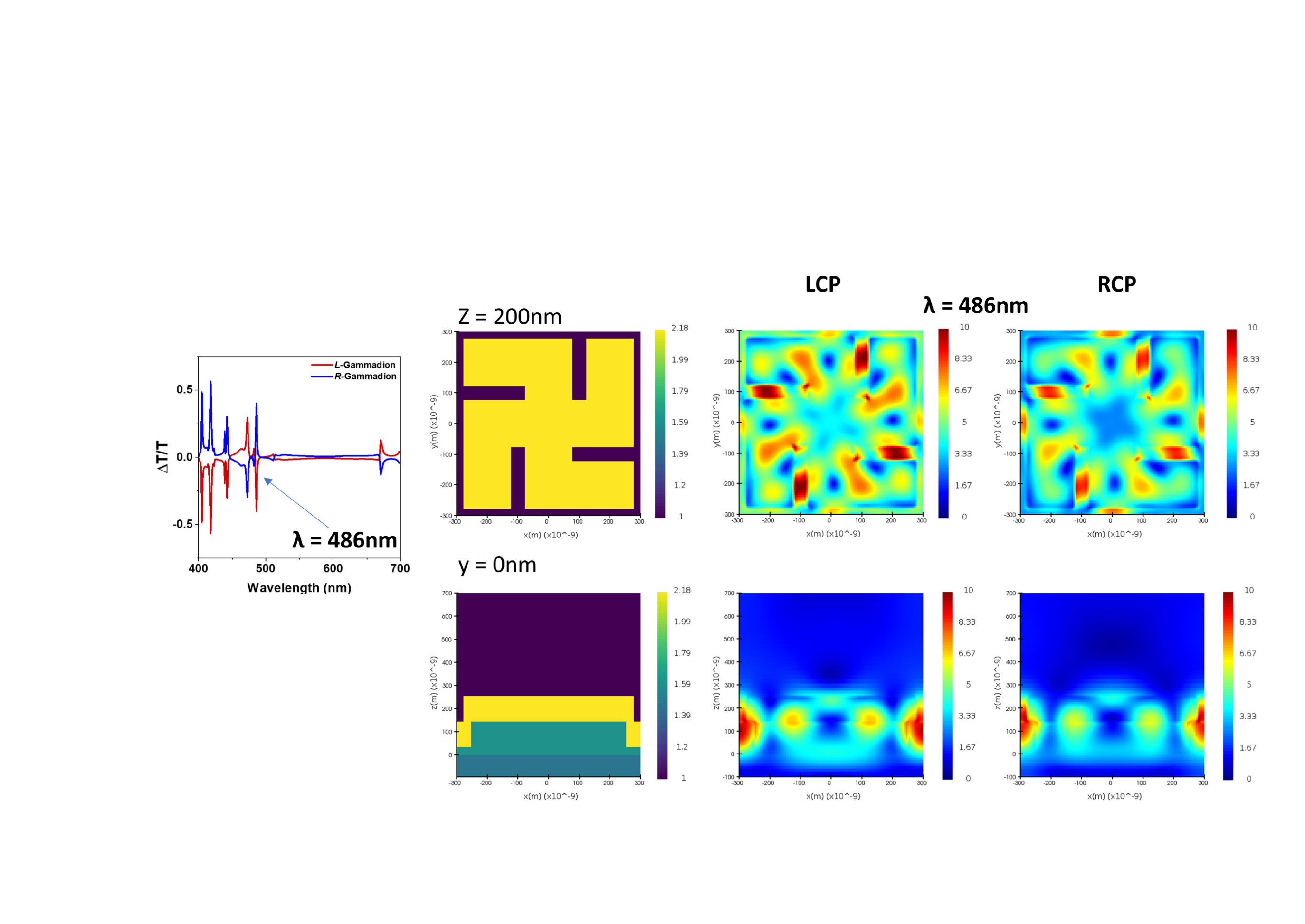


**Figure S9.** Simulated CD transmission characterization of the 100 nm TiO_2_ coated *L*-and *R-*Gammadions.

Simulations for gammadions with 100 nm TiO_2_ are shown below. For this, we have focused on the 486 nm wavelength. In the transmission characterization spectrum, the effective blueshifted structures can be seen compared to those obtained experimentally, because they lack the effective medium of perovskites on top that would shift the resonances toward the red (**Figure S8**). After analyzing the electric fields (**Figure S9**), it seems that the higher LCP in-coupling generates hotspots between the gammadion arms where the perovskites are located, generating a more intense mode that translates into higher RCP out-coupling (negative g_lum_).


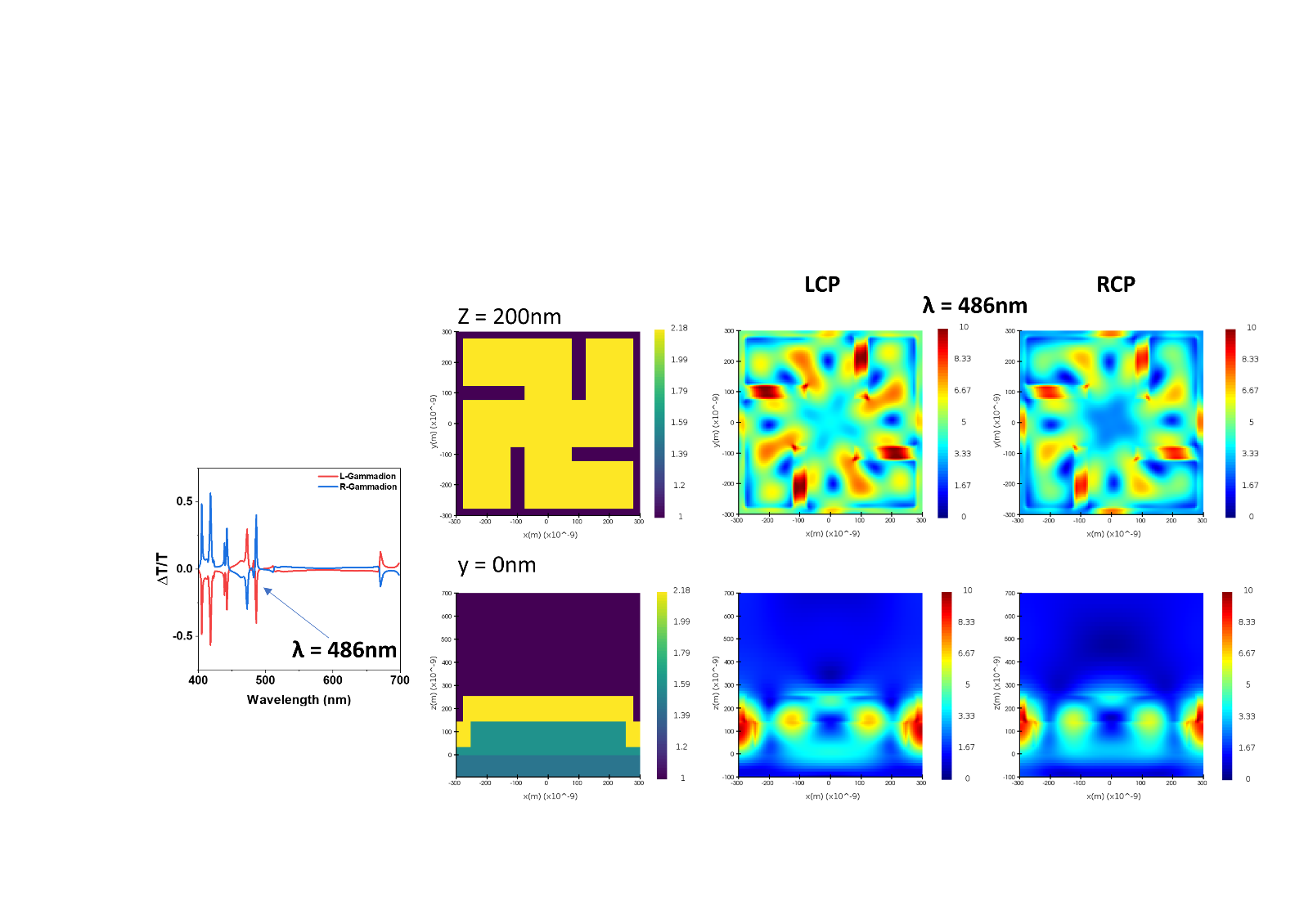


**Figure S10.** Simulations of the electric field of the TiO_2_-coated *L-*Gammadion.

After performing a simulation to study the effect of the magnetic component changes (**Figure S11**), we can see in the same way, that it seems that the higher in-coupling of LCP generates a more intense mode which translates into higher out-coupling of RCP leading to a negative g_lum_.


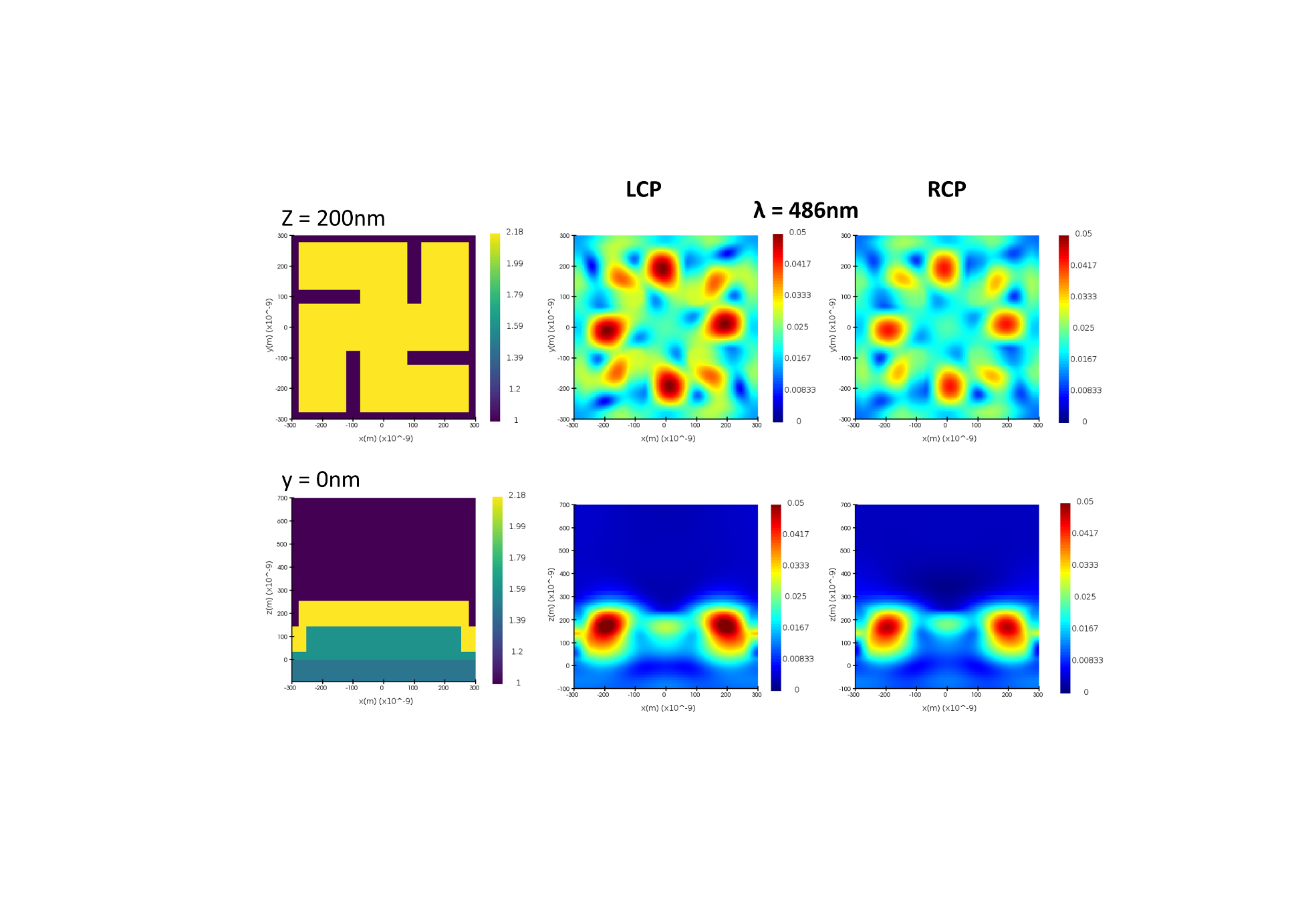


**Figure S11.** Simulations of the magnetic field effect in the 100 nm TiO_2_ coated *L-*Gammadion.

The resonances obtained by simulating gammadions coated with 25 nm of gold coincide with those obtained experimentally, with their most intense values at around 650 nm (**Figure S12**).


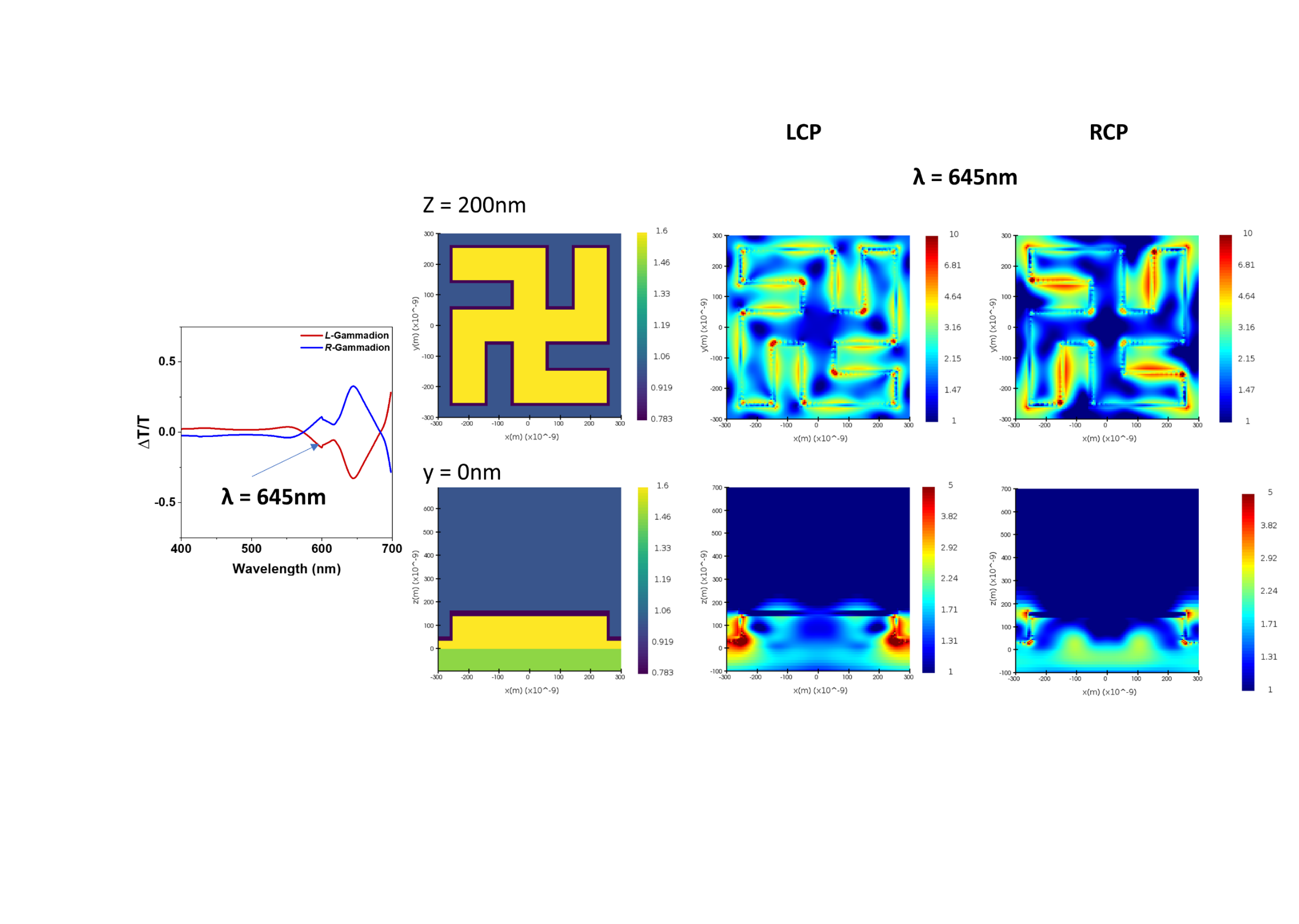


**Figure S12.** Simulated transmission CD characterization for *L-* and *R-* Gammadions coated with 25 nm Au.

In the simulations performed for the 25 nm gold-coated gammadion, the light injection is from the glass. Therefore, the *L-*Gammadion has higher LCP in-coupling, so there is higher RCP outcoupling from the glass side, resulting in a negative g_lum_.


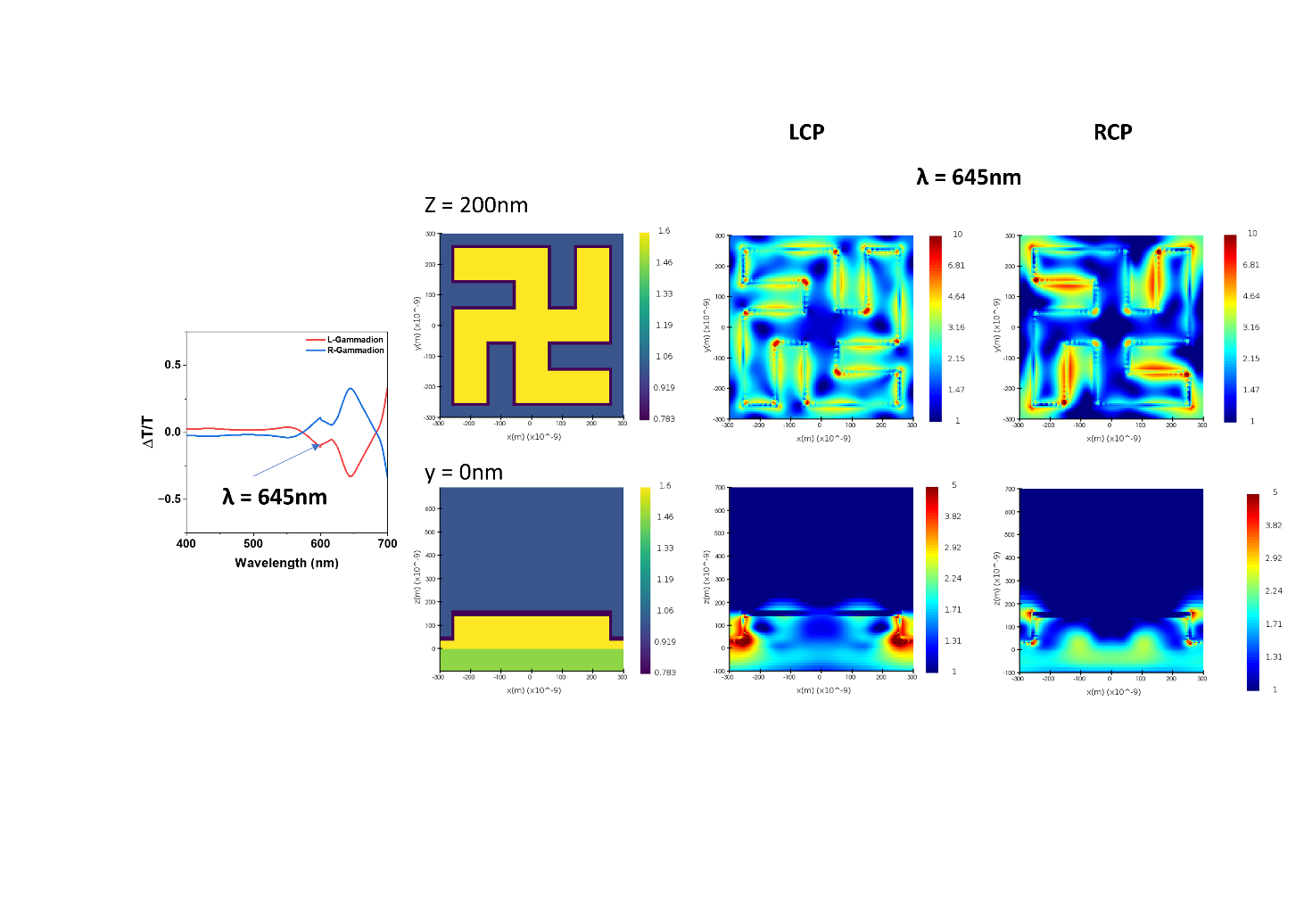


**Figure S13**. Simulations obtained for 25 nm coated *L-*Gammadion at 645 nm.

To unravel the differences between the dielectric and plasmonic-coated metasurfaces, we simulate a periodic array of *L-*Gammadion coated with these materials and compute the fraction of reflected and transmitted power. Then, we can compute the extinction of the metasurface extracted from the total reflectance and transmittance as:

| $E = 1-R-T$ | (6) |
| --- | --- |

We simulate an SU8 L = 500 nm wide *L-*Gammadion, with w = 100nm wide arms and of h = 110 nm tall atop of a r = 30 nm residual flat layer. Then, it is coated with either t = 100 nm of TiO_2_ or t = 25 nm of Au, and disposed with periodic boundary conditions of lattice Λ= 600 nm. We inject into the system two circular polarizations by combining linearly polarized x and y plane waves and introducing a phase delay between these of ±π/2. **Figure S14** summarizes all these results for the dielectric TiO_2_ (top row) and the plasmonic Au coating (bottom row). We included colored shaded areas in blue (475 nm), green (520 nm), and red (650 nm) to indicate the emission bands of the perovskite NCs used herein. In the dielectric case, we observe sharp diffractive resonances along the blue and green regions, overlapping with the emission bands of perovskites in both transmittance (**a**) and reflectance (**b**). This can be seen in the extinction spectrum (**c**) where these excited surface modes that do not reach either of the detectors can be seen as extinction peaks overlap with the blue and green emission bands. As for the red-emitting case, even though the emission band is slightly overlapped with a resonant mode, there are no signatures in the extinction spectrum, thus hindering the CPE arising at this wavelength. As for the plasmonic coating, we observe that blue and green emission bands do not overlap regions where chirality is present neither in reflectance (**d**) or transmittance (**e**). On the other hand, the red-emitting perovskite overlaps with a differential transmittance region. Therefore, when we investigate the extinction coefficient, we observe differential absorption rates for each polarization at the red-emitting wavelength, but not for the blue or green ones (**f**). Based on these results, we can conclude that the main phenomena driving CPL for the dielectric case is diffraction assisted, scattering off-normal one of the polarizations^[8]^ , whereas for the plasmonic case, differential absorption is the main responsible for inducing the polarization conversion in normal direction.^[9]^


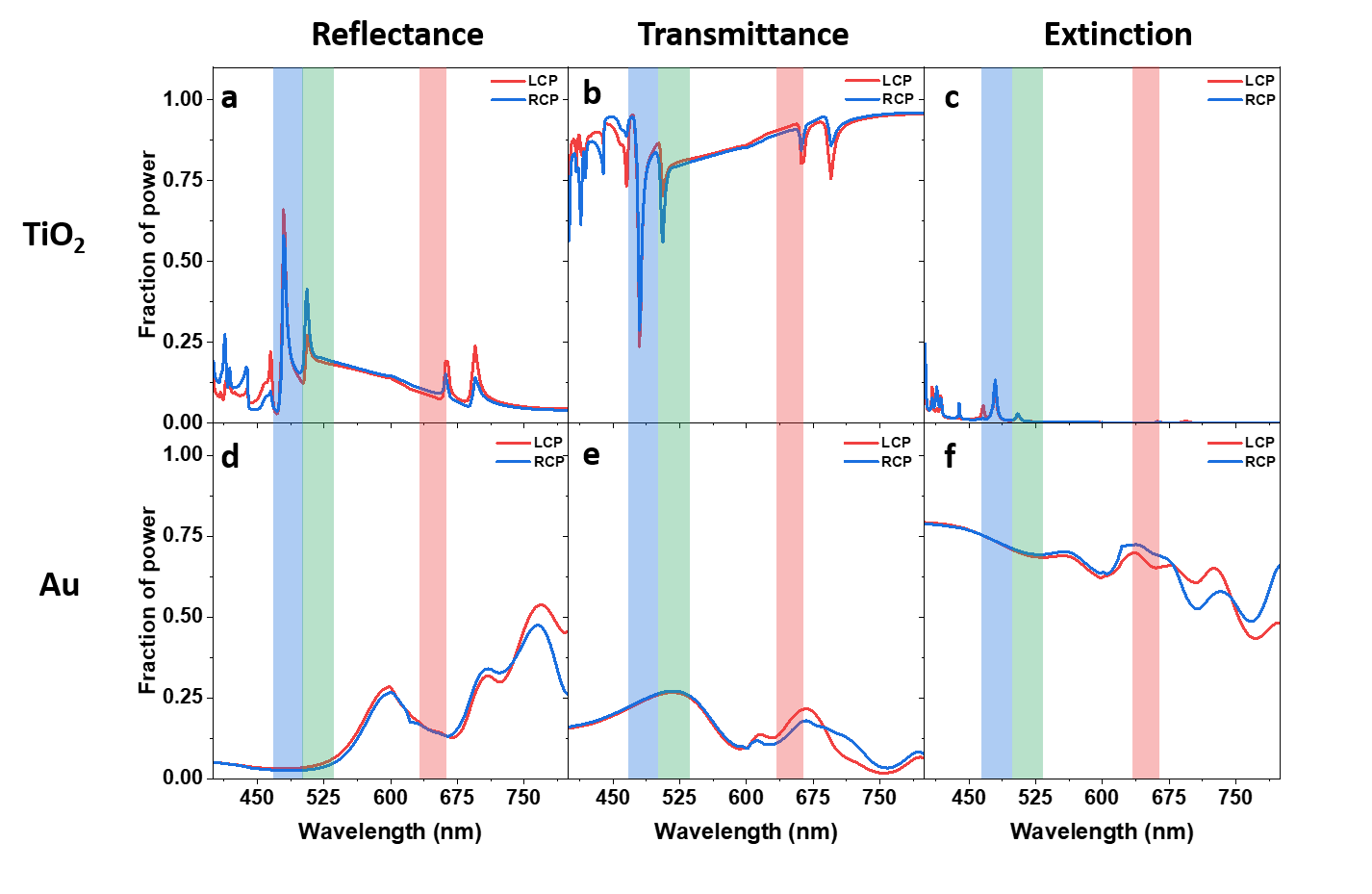


**Figure S14.** (a) Reflectance (b) Transmittance and (c) Extinction spectra of TiO_2_ chiral metasurfaces. (d) Reflectance (e) Transmittance and (f) Extinction spectra of Au chiral metasurfaces overlapping with the blue, green or red emission bands of the NCs (shaded area).

**Supplementary Information 9. Emission Spectra Obtained for 25 nm Coated Au Gammadions with Perovskite NCs of Different Compositions**


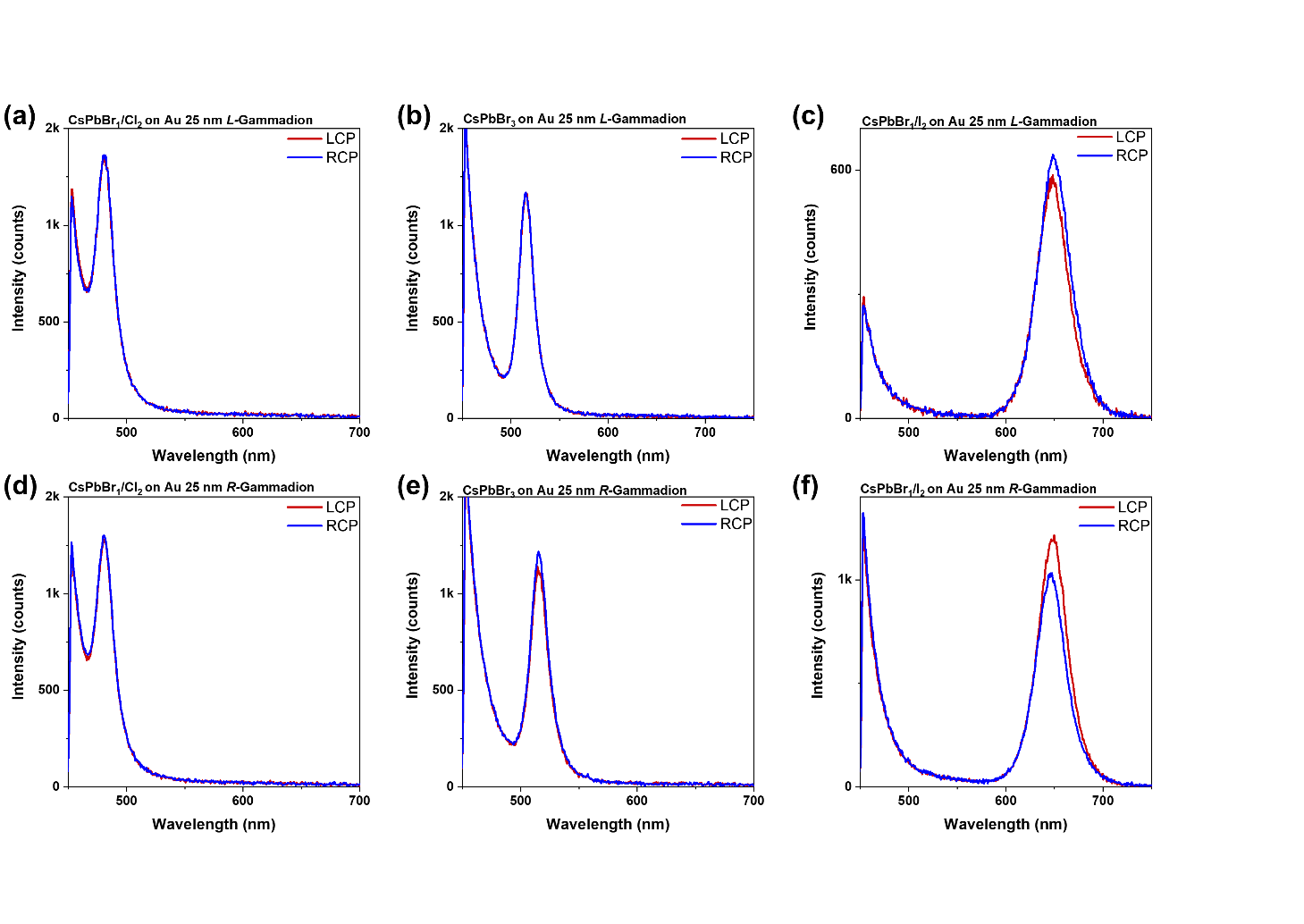


**Figure S15.** CPL Spectra for *L-*/*R-*Gammadions with CsPbBr_1_Cl_2_ NCs (a,d), CsPbBr_3_ (b,e) and CsPbBr_1_I_2_ 25 nm Au coated gammadions.

**Supplementary Information 10. Unraveling Possible PL Quenching Effects Depending on the Coating Material**

In this section, we study the possible PL quenching effects that can occur to the emitters in the vicinity of the coating material. For the dielectric TiO_2_ metasurfaces, as no absorption process overlaps with the emission bands, we do not expect quenching processes to occur in the PL. On the other hand, metallic metasurfaces are widely known to suffer from quenching effects, as these structures can sustain surface plasmon polaritons matching the emission wavelength. However, there are many works in which plasmon resonances, particularly diffraction-coupled far-field resonances, are used to enhance light emission.^[10-12]^ Aiming to unravel the effect of the material, we compare the difference PL intensity of the emitters both from the metasurface and the unpatterned area for both materials. As shown in **Figure S16a**, we obtain a two-fold enhancement on the measured PL intensity when measuring inside the pattern, which confirms the enhanced out-coupling efficiency driven by diffraction effects present in TiO_2_. On the contrary, in the case of Au gammadions (**Figure S16b**), diffraction processes are hindered by absorption, and therefore the light extraction is not as efficient as in the dielectric case, showing similar PL intensity values for both areas.


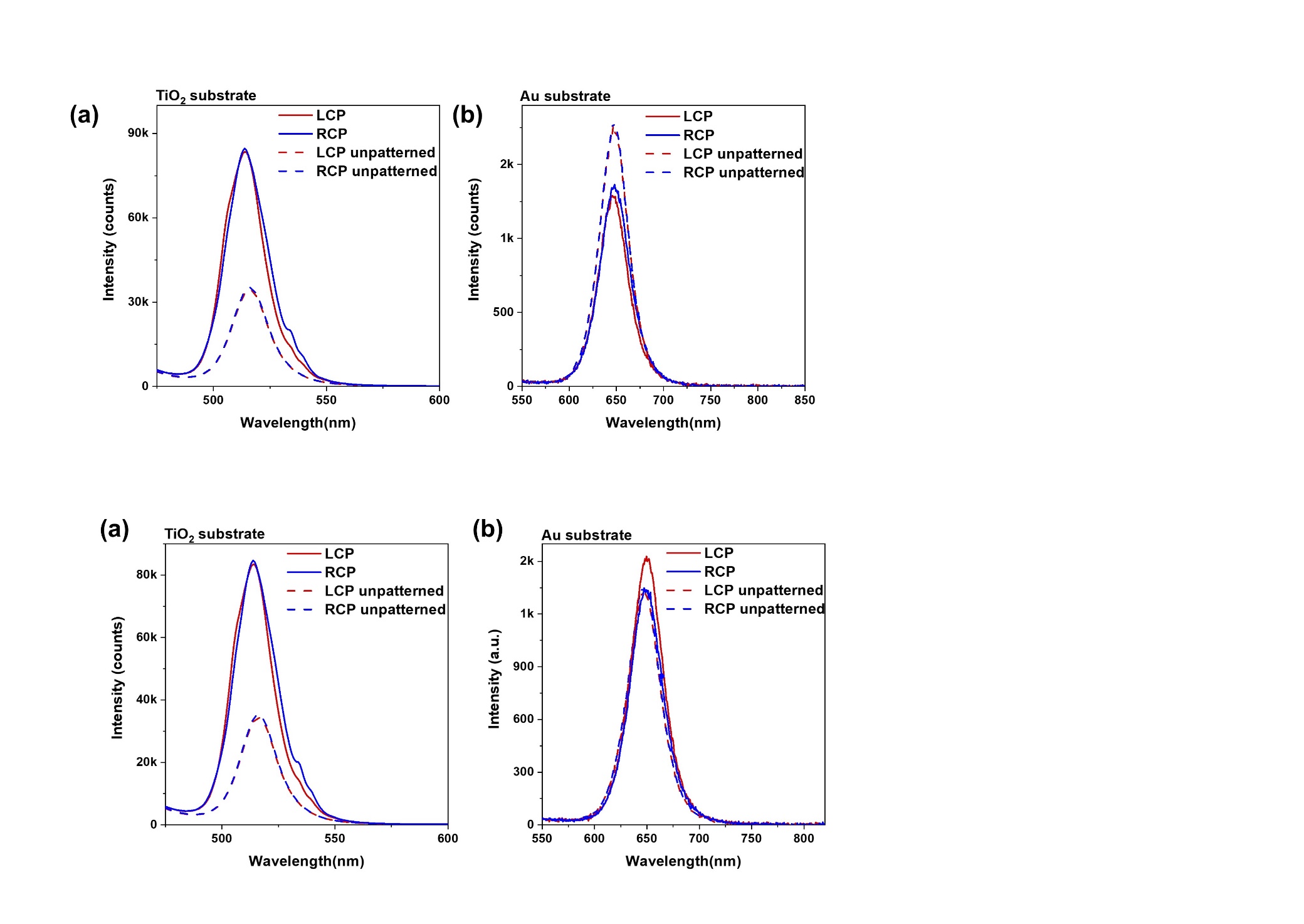


**Figure S16.** (a) PL Spectra for TiO_2_ *L-*gammadions substrate measured inside the pattern and out of the pattern (dotted line). (b) PL Spectra for Au *L-*gammadion substrate measured inside the pattern and out of the pattern (dotted line)

**Supplementary Information 11. Transmission CD of Hybrid Structures**

The following figure shows the comparison in transmittance CDs of the TiO_2_-gold hybrid structures with coupled or uncoupled gammadions. **Figure S17 a,b** show a hybrid structure that is coupled, consisting of a double coating of TiO_2_ followed by a gold coating. **Figure S17 c,d** correspond to the uncoupled hybrid structures, manufactured with a gold-coated imprint followed by a second imprint at a distance that allows their characteristic resonances to be preserved.


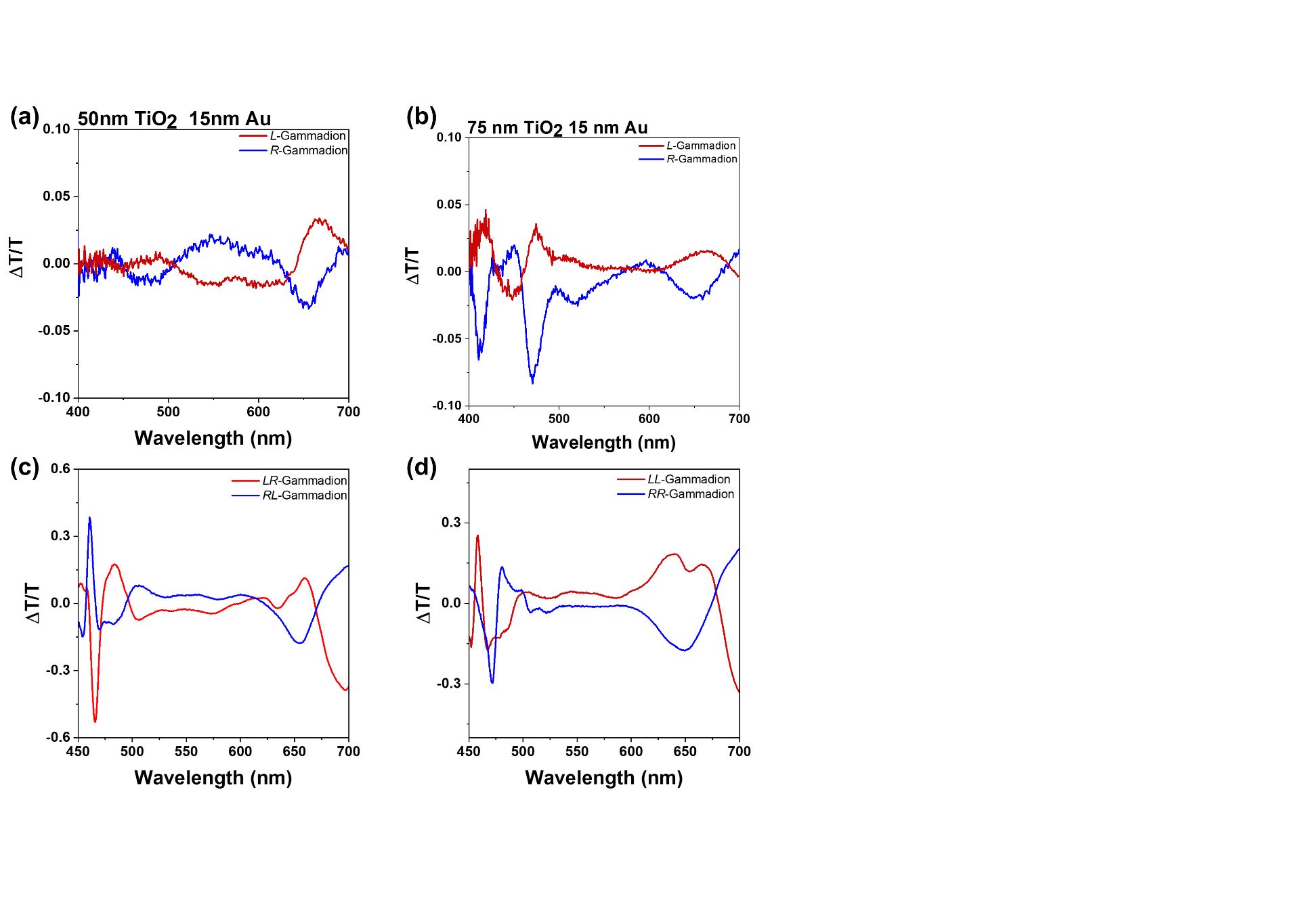


**Figure S1*7*.** Transmission CD Spectra of Hybrid Structures. a) *L-* and *R-* Gammadion coated with 50 nm of TiO_2_ followed by 15 nm Au. b) *L-* and *R-* Gammadion coated with 75 nm of TiO_2_ followed by 15 nm Au. c-d) Transmission CD with different handedness combinations, where the first letter refers to the first imprint and the second one to the second imprint.

**Supplementary Information 12. PL Spectra Characterization of Racemic Hybrid Structure**

CPL obtained for racemic Au/TiO_2_-coated Hybrid Gammadions with the addition of CsPbI_2_Br_1_ PVK NCs on the backside of the sample and CsPbBr_3_ on the top.


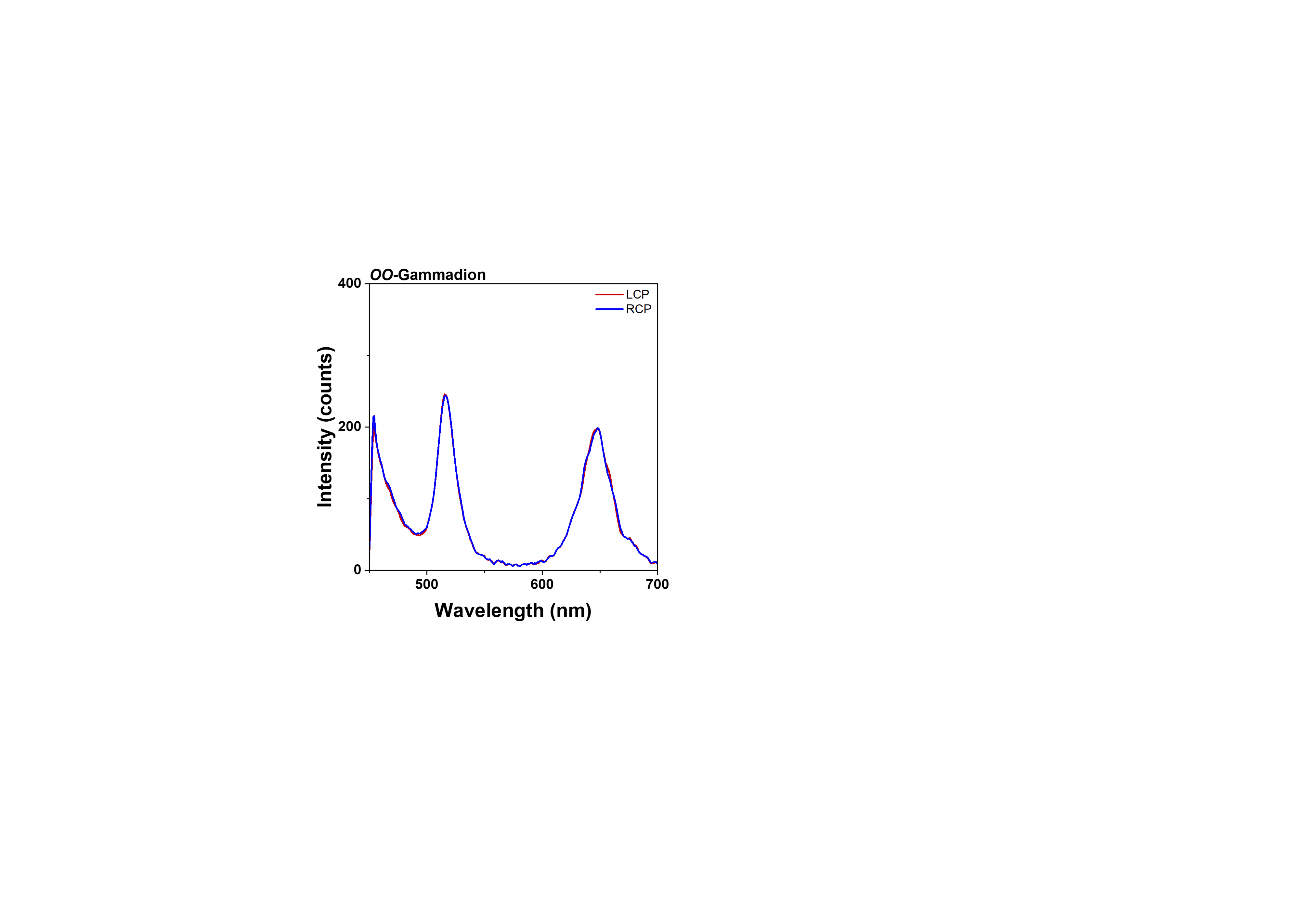


**Figure S18.** CPL of hybrid structures of TiO_2_ and Au of the racemic control structure*.*

**References**

[1] M. J. S. S. i. O. S. Schäferling, **2017**, 205, 159.

[2] X. Ma, M. Pu, X. Li, Y. Guo, P. Gao, X. J. N. Luo, **2017**, 7, 116.

[3] G. Long, G. Adamo, J. Tian, M. Klein, H. N. S. Krishnamoorthy, E. Feltri, H. Wang, C. Soci, *Nature Communications* **2022**, 13, 1551.

[4] Y. Tang, A. E. J. P. r. l. Cohen, **2010**, 104, 163901.

[5] C.-L. Yu, Y.-H. Hsiao, C.-Y. Chang, P.-J. Cheng, H.-T. Lin, M.-S. Lai, H.-C. Kuo, S.-W. Chang, M.-H. Shih, *Scientific Reports* **2020**, 10, 7880.

[6] J. Tian, G. Adamo, H. Liu, M. Klein, S. Han, H. Liu, C. Soci, **2022**, 34, 2109157.

[7] J. Mendoza-Carreño, P. Molet, C. Otero-Martínez, M. I. Alonso, L. Polavarapu, A. Mihi, **2023**, 35, 2210477.

[8] A. Y. Zhu, W. T. Chen, A. Zaidi, Y.-W. Huang, M. Khorasaninejad, V. Sanjeev, C.-W. Qiu, F. Capasso, *Light: Science & Applications* **2018**, 7, 17158.

[9] E. S. A. Goerlitzer, A. S. Puri, J. J. Moses, L. V. Poulikakos, N. Vogel, **2021**, 9, 2100378.

[10] R. Faggiani, J. Yang, P. J. A. p. Lalanne, **2015**, 2, 1739.

[11] J. Yang, R. Faggiani, P. J. N. h. Lalanne, **2016**, 1, 11.

[12] V. Kravtsov, S. Berweger, J. M. Atkin, M. B. J. N. l. Raschke, **2014**, 14, 5270.
